# Single-cell heterogeneity in ribosome levels and protein synthesis during nutrient starvation is driven by cAMP signaling

**DOI:** 10.1126/sciadv.aed2171

**Published:** 2026-06-26

**Authors:** Zhihui Lyu, Jacques Augenstreich, Volker Briken, Abhyudai Singh, Matteo Mori, Terence Hwa, Jiqiang Ling

**Affiliations:** ^1^ Department of Cell Biology and Molecular Genetics, The University of Maryland, College Park, MD 20742, USA.; ^2^ Department of Electrical and Computer Engineering and Biomedical Engineering, University of Delaware, Newark, DE 19716, USA.; ^3^ Department of Physics, University of California at San Diego, La Jolla, CA 92093, USA.

## Abstract

Ribosomes are central to protein synthesis and a frequent target for antibiotics. In fast-growing bacteria, the ribosome content is proportional to the growth rate; how ribosomes and protein synthesis are regulated during nutrient starvation remains poorly understood, particularly in single cells. To address this, we fluorescently labeled ribosomal proteins (RPs) in *Salmonella* and explored their variations and regulation in single cells. We show that the RP levels become heterogeneous during the transition to the stationary phase. Unexpectedly, cells with higher RP levels responded less to the induction of gene expression but accumulated more virulence gene products. Our work further reveals that adenosine 3′,5′-monophosphate (cAMP) signaling increases the heterogeneity of the levels of RPs and other gene products. Fluorescence dilution assay and proteomic analysis indicate that cAMP signaling promotes gene expression heterogeneity by directing proteome-wide adaptation, which enables growth heterogeneity, hence differential dilution of gene products during nutrient depletion.

## Teaser

Cell signaling promotes differential growth and variations in ribosome levels and protein synthesis.

## INTRODUCTION

Protein synthesis is a central cellular process involving the production of aminoacyl-tRNAs by aminoacyl-tRNA synthetases and decoding of mRNA codons by matching aminoacyl-tRNAs on the ribosome, a macromolecular machinery composed of more than 50 ribosomal proteins (RPs) and 3 or 4 ribosomal RNAs (rRNAs) (*1*–*3*). Ribosome biogenesis and protein synthesis are energetically costly and highly regulated (*4*). At the population level, ribosome abundance linearly increases with the growth rate (*4*–*6*), while it declines during starvation (*4*–*7*), where synthesis of RPs and rRNAs is repressed by the stringent response mediated by (p)ppGpp through the transcription factor DksA (*8*–*10*). Ribosome abundance has been shown to vary substantially from cell to cell already during exponential growth (*11*) and also during starvation (*12*); possible causes of such variations, as well as their physiological impact, are poorly understood.

Phenotypic heterogeneity among single cells is critical for bacterial populations to survive stresses and adapt to changing environments and is often a manifestation of an underlying bet-hedging strategy or division of labor (*13*–*18*). Variations in gene expression and amplification by positive feedback among individual cells are common mechanisms driving phenotypic diversity (*14*, *19*–*22*). Previous single-cell studies have used the promoter activities of rRNA operons to estimate ribosome abundance (*23*, *24*). While this approach has useful applications, it does not directly measure the cellular ribosome level. Recent studies have also used fluorescence in situ hybridization (FISH) to detect rRNAs in single cells, revealing substantial heterogeneity in the rRNA content among single bacterial cells (*12*, *25*). However, FISH requires cell fixation and is not suitable for studying live cells; furthermore, it does not readily lend itself to cross-correlating with gene expression levels in the same cells. The hybridization efficiency of rRNA FISH can also be affected by RNA-RNA and RNA-protein interactions within the ribosome (*26*). Alternatively, labeling of RPs with fluorescent proteins has been used to study the localization and abundance of ribosomes in *Escherichia coli* (*11*, *27*–*30*). Previous studies, involving the labeling of the peripheral 50S protein bL19 and 30S protein uS2 in *E. coli* (*31*, *32*), have shown that these RPs provide a good estimate of the assembled ribosomes while not affecting ribosome function and cell growth (*31*, *32*). However, these studies largely focused on exponentially growing cells at the population level.

In nature, bacterial cells frequently encounter nutrient limitations and enter a quiescent state loosely referred to as the “stationary phase” as nutrients become depleted (*7*). Stationary-phase cells display increased tolerance to stresses and antimicrobials, associated with the expression of stress proteins such as those driven by the general stress σ factor RpoS (*7*, *16*, *18*, *33*, *34*). Targeting quiescent pathogens is key to eliminating persister cells in successful antimicrobial treatments (*35*, *36*). Upon transitioning to the stationary phase, cells remain metabolically active (*7*, *21*). How the RP levels and gene expression are regulated in stationary-phase cells remains an open question. In this work, we fluorescently labeled bL19 and uS2 in *Salmonella enterica* Typhimurium and observed a substantial increase in the heterogeneity of RP levels during the transition from exponential growth to the stationary phase in rich media. Deleting DksA, which reduces the repressive effect of (p)ppGpp on ribosomal expression (*9*, *12*), increased the mean RP levels in single cells and decreased their variations. Deleting CyaA, which abolishes the synthesis of adenosine 3′,5′-monophosphate (cAMP) (*37*–*39*), a second messenger well known for its role in up-regulating carbon catabolism (*40*, *41*), also decreased RP variations. These findings point to the critical roles that the CyaA-mediated cAMP signaling system and the DksA-mediated (p)ppGpp signaling systems play in sustaining ribosome variations in single cells during the stationary phase. Our data further suggest that cAMP signaling contributes to RP variations in wild-type (WT) cells by altering their growth characteristics during the course of nutrient depletion, resulting in a major subpopulation of cells retaining more ribosomes because of their lack of growth-mediated dilution when growth is abruptly halted. In addition, we found that cells with higher RP levels responded less to the induction of gene expression during the stationary phase but accumulated more products from virulence genes during growth slowdown. Proteomic analysis of samples taken from the exponential and stationary phases for WT and Δ*cyaA* and Δ*dksA* mutants suggests a scenario in which an abrupt growth arrest of WT cells results from proteome-wide adaptation guided by cAMP signaling as nutrients deplete during transition to the stationary phase.

## RESULTS

### Increased heterogeneity of RP levels during the transition from growth to quiescence

**Fig. 1.**
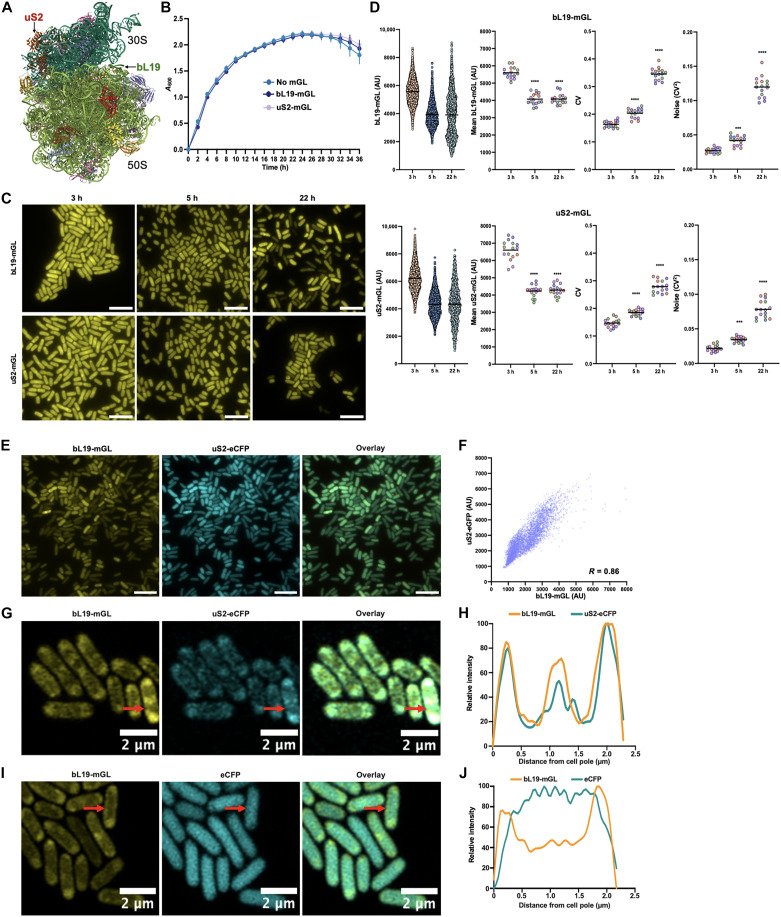
Fluorescence labeling and colocalization of RPs. (**A**) Cryo–electron microscopy structure of *E. coli* ribosome [PDB (Protein Data Bank): 7K00] (*84*). uS2 and bL10 are peripheral RPs in the 30S and 50S subunits, respectively. h, hours. (**B**) Growth of *Salmonella* Typhimurium strains in LB at 37°C. bL19-mGL and uS2-mGL are chromosomally tagged with mGL fluorescent protein. Error bars represent one standard deviation from the means (*n* = 8). (**C**) Representative fluorescence microscopy images of *Salmonella* cells at various growth stages. (**D**) Quantitation of fluorescence signals in individual cells and their heterogeneity among cells. The left-most panels show a representative fluorescence plot of individual cells. Each dot represents one cell. In the other panels, biological replicates are indicated in different colors, and technical replicates are shown in the same colors. AU, arbitrary units. (**E**) Dual fluorescence labeling of bL19 and uS2 on the chromosome with mGL and eCFP, respectively. *Salmonella* cells were grown to the stationary phase (22 hours). (**F**) Correlation between bL19-mGL and uS2 eCFP signals in (E). Each dot represents one cell. (**G** and **I**) Superresolution imaging of dual-labeled *Salmonella* cells grown to the stationary phase. (**H** and **J**) Representative subcellular distribution of fluorescence intensity in the cells indicated by arrows in (G) and (I). The *P* values are calculated using the one-way ANOVA with Dunnett’s test compared with the 3-hour sample. ***P < 0.001, ****P < 0.0001. Scale bars in (C) and (E), 5 μm.

A recently developed fluorescent protein, mGreenLantern (mGL), is bright, fast-maturing, and monomeric, making it well suited for single-cell analyses (*42*). To quantify RP levels in single cells, we constructed bL19-mGL and uS2-mGL *Salmonella* strains by chromosomally fusing the *mGL* coding region in-frame at the 3′-end of the native *rplS* and *rpsB* genes, which encode bL19 and uS2, respectively (Fig. 1A and tables S1 and S2). Fluorescence labeling of bL19 and uS2 resulted in the expected sizes of the fusion proteins (fig. S1) and did not affect *Salmonella* growth in Luria-Bertani Lennox broth (LB) at 37°C (Fig. 1B). Time-course analyses revealed that bL19-mGL and uS2-mGL were stable under this growth condition (fig. S2). The polysome profiles of bL19 cells revealed that the ribosomes were appropriately assembled (fig. S3A).

We next performed fluorescence microscopy imaging of mGL-labeled cells grown to the exponential (3 hours), transition (5 hours), and stationary (22 hours) phases (Fig. 1C). Quantitation of single-cell fluorescence revealed that the mean fluorescence of bL19-mGL and uS2-mGL decreased at 5 and 22 hours compared with 3 hours (Fig. 1D), reflecting the expected decrease in ribosome levels as growth slows down (*6*). In line with this, we observed a decrease in the RNA/protein ratio at 22 hours (fig. S3B), which has been used as a proxy for the rRNA content (*43*, *44*). The mean cell sizes were also lower during the transition and stationary phases compared with the exponential phase (fig. S4), reflecting the expected decrease in cell size as growth slows down (*45*). Intriguingly, the heterogeneity [indicated by the coefficient of variance (CV)] of RP levels began to increase at 5 hours and substantially increased at 22 hours (Fig. 1D). Almost all cells were viable at 22 hours as they were able to regrow when fresh medium was supplied (fig. S5). However, over longer periods of culturing, bL19 and uS2 began to form foci in cells (fig. S6). We thus focused this study on the transition to the stationary phase during the first 22 hours.

### Colocalization of bL19 and uS2 in the stationary phase

Previous work has shown that *E. coli* cells contain equimolar amounts of different RPs in the stationary phase (*46*). To test the correlation between RP levels in single *Salmonella* cells, we dual labeled bL19 and uS2 with mGL and enhanced cyan fluorescent protein (eCFP), respectively. mGL and eCFP exhibited negligible background and did not cross-talk under our fluorescence microscopy conditions (fig. S7). At 22 hours, the levels of bL19-mGL and uS2-eCFP were strongly correlated among single cells (Fig. 1, E and F). Further superresolution microscopy analyses revealed that bL19-mGL and uS2-eCFP colocalized at the subcellular level (Fig. 1, G and H). In contrast, no colocalization was observed between bL19-mGL and free eCFP (Fig. 1, I and J). bL19-mGL and uS2-eCFP were mostly enriched at the cell poles and occasionally in the mid-cell, consistent with the notion that bacterial chromosomes exclude 70S ribosomes (*27*). The cells with ribosomes enriched in the middle likely contained separated chromosomes. In addition, our polysome profile revealed that most ribosomes were 70S monosomes at 22 hours (fig. S3A). These results support the notion that bL19 and uS2 mostly exist in assembled ribosomes, consistent with previous work in *E. coli* (*31*, *32*).

### Ribosome hibernation does not affect RP variations

Stationary-phase bacterial cells use several hibernation factors to protect ribosomes (*47*, *48*). The ribosome modulation factor (encoded by the *rmf* gene) initiates a process to form the 100S hibernating ribosome from two 70S ribosomes (*47*). In addition, RaiA (ribosome-associated inhibitor A) binds to the 70S ribosome and inhibits its activity (*48*). To test whether ribosome hibernation affects the levels of RPs in single cells, we deleted *rmf* and *raiA* in the bL19-mGL strain individually and in combination. None of the resulting mutants exhibited a significant change in the mean bL19-mGL level or its heterogeneity (fig. S8), suggesting that RMF (ribosome modulation factor) and RaiA do not play a major role in the variations of bL19 levels. This is in line with our time-course results showing that bL19 is stable during the transition and stationary phases (fig. S2). The RNA/protein ratio in the Δ*rmf*/*raiA* cells was lower compared to the WT (fig. S8C). It is possible that deleting *rmf* and *raiA* makes the ribosome less stable in the stationary phase and promotes the degradation of rRNAs, whereas the levels of RPs are less affected.

### cAMP enhances RP variations

**Fig. 2.**
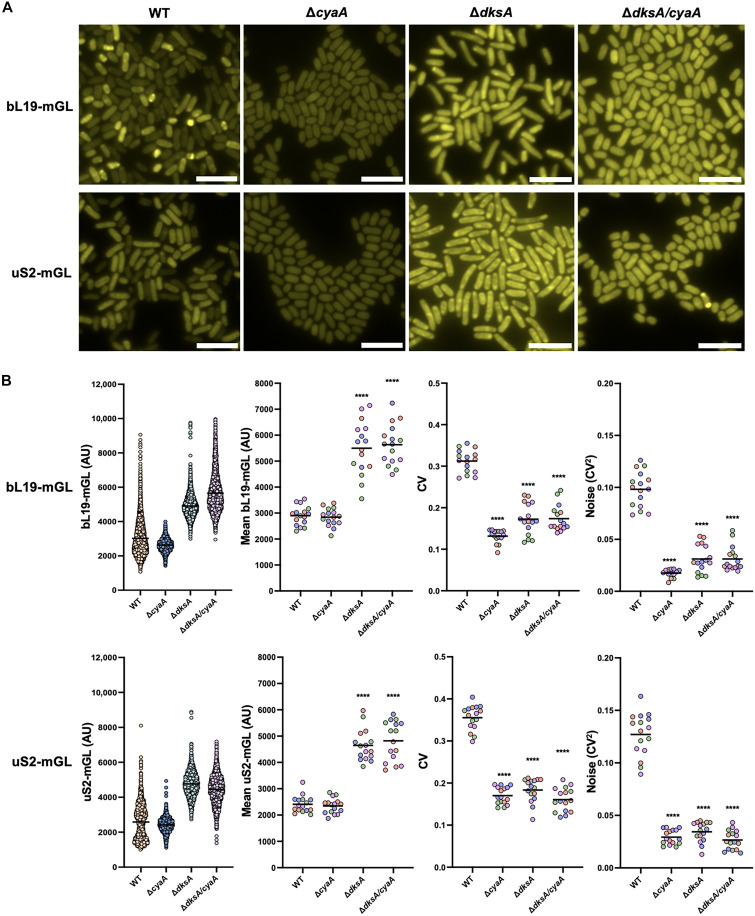
CyaA and DksA promote heterogeneity in RP levels. (**A**) Representative fluorescence microscopy images of *Salmonella* cells grown to the stationary phase (22 hours). (**B**) Quantitation of fluorescence signals in individual cells and their heterogeneity. Biological replicates are indicated in different colors, and technical replicates are shown in the same colors. The *P* values are calculated using the one-way ANOVA with Dunnett’s test comparing mutants with the WT. ****P < 0.0001. Scale bars, 5 μm.

cAMP is a key regulator of carbon source utilization and allocation of proteomic resources (*37*, *38*). Our recent work demonstrates that cAMP enhances the heterogeneity of the rRNA promoter activity (*24*), prompting us to investigate the role of cAMP in RP variations. In *E. coli* and *Salmonella*, the only enzyme that synthesizes cAMP is encoded by *cyaA* (*37*–*39*). We found that deleting *cyaA* significantly reduced the heterogeneity of bL19 and uS2 levels at 22 hours by virtually removing the high-RP cell fraction without affecting the peak of the distribution (Fig. 2). Complementation of the *cyaA* gene or addition of cAMP in the medium restored the heterogeneity of bL19-mGL in the Δ*cyaA* strain to the WT level (figs. S9 and S10). cAMP modulates the activity of cAMP receptor protein (CRP) to regulate gene expression (*40*, *41*). Deleting *crp* caused a similar decrease in the CV of bL19-mGL as deleting *cyaA* (fig. S11), suggesting that the effect of cAMP on RP variations is via the cAMP/CRP pathway.

**Fig. 3.**
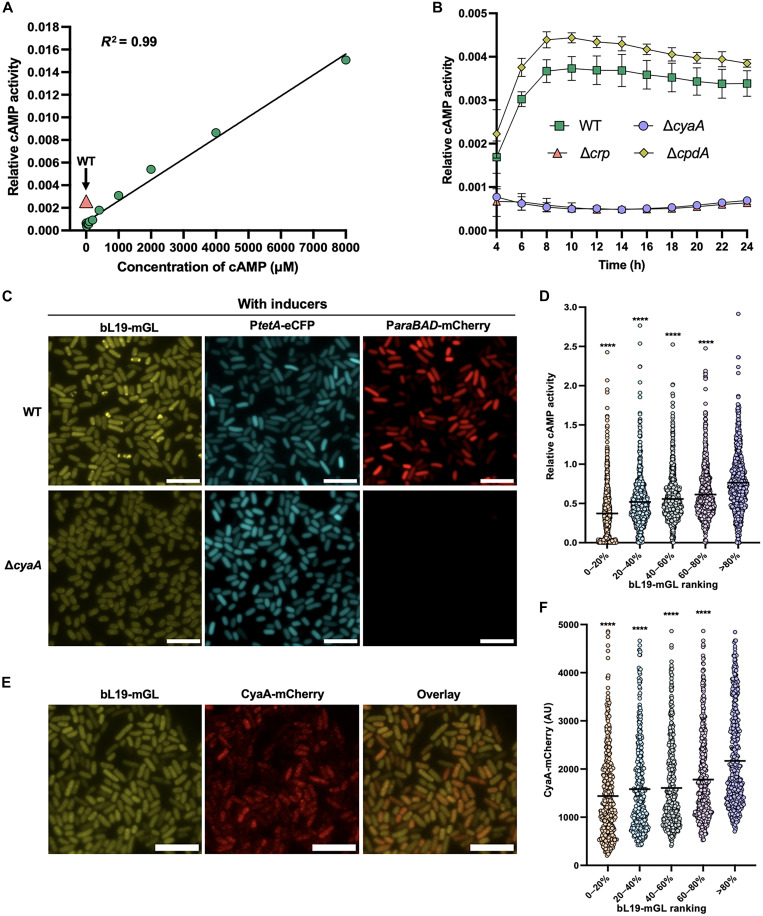
Inducible dual-reporter system for cAMP activity. (**A**) pZS P*tetA-*eCFP P*araBAD*-mCherry was expressed in the WT bL19-mGL strain as a reporter for the relative cAMP activity, calculated as the mCherry/eCFP ratio. P*tetA* and P*araBAD* were induced with aTc (250 ng/ml) and 1 mM l-arabinose, respectively. (A) Titration of relative cAMP activities using a plate reader. Cells were grown in LB at 37°C for 4 hours with inducers and various concentrations of added cAMP, which can enter the cells. In Δ*cyaA* cells, the relative cAMP activity linearly correlates with the cAMP concentration in the medium. (**B**) Relative cAMP activities over time. (**C**) Representative fluorescence images of stationary phase cells (22 hours). aTc (250 ng/ml) and 1 mM l-arabinose were added at 5 hours. In the absence of *cyaA*, no mCherry was observed with the addition of l-arabinose, suggesting that P*araBAD* activity is dependent on cAMP. (**D**) Bin analysis of relative cAMP activities in (C) based on the bL19-mGL ranking from low to high. (**E**) Representative fluorescence images of bL19-mGL and CyaA-mCherry dual-labeled cells grown in LB for 22 hours. (**F**) Bin analysis of (E). The *P* values are calculated using the one-way ANOVA with Dunnett’s test comparing other subpopulations with the high-bL19 subpopulation (>80%). The images and bin analyses are representatives of at least three biological replicates. ****P < 0.0001. Scale bars, 5 μm.

To measure the cAMP activity in single cells, we constructed a dual-inducible promoter reporter system with eCFP and mCherry controlled by P*tetA* (*49*) and P*araBAD*, respectively (Fig. 3). P*tetA* is strictly controlled by anhydrotetracycline (aTc) (Fig. 3C and fig. S12). In the presence of the inducer l-arabinose, the *araBAD* promoter is controlled by cAMP in *E. coli* (*50*) and in *Salmonella* (Fig. 3). Using the ratio of mCherry/eCFP in the presence of inducers, we found that the relative cAMP activity in the Δ*cyaA* strain linearly correlated with the concentration of cAMP added to the medium (Fig. 3A), suggesting that our duo-reporter system is quantitative for the cAMP activity. The WT cells exhibited a relative cAMP activity similar to that of the Δ*cyaA* cells with 1 mM added cAMP. Deleting *cpdA*, which encodes the cAMP phosphodiesterase, slightly increased the cAMP activity (Fig. 3B).

We next used the dual-reporter system to measure cAMP activities in single WT and Δ*cyaA* cells during the transition to the stationary phase (Fig. 3, C and D). Deleting *cyaA* abolished P*araBAD*-mCherry signals even in the presence of l-arabinose (Fig. 3C). WT cells with the highest bL19-mGL levels also exhibited the highest cAMP activity (Fig. 3D). To further investigate how cAMP contributes to RP variations, we tested bL19-mGL fluorescence in the Δ*cpdA* strain. Deleting *cpdA* did not significantly affect the CV of bL19-mGL (fig. S11), suggesting that degradation of cAMP is not a major factor for the heterogeneity of cAMP activity and RP levels. To test the CyaA levels in single cells, we constructed a translational *cyaA-mCherry* fusion at the native *cyaA* site in the bL19-mGL strain. The CyaA-mCherry levels were low and heterogeneous in the stationary phase (Fig. 3E). The levels of CyaA-mCherry and bL19-mGL were also positively correlated among single cells (Fig. 3F). Our results suggest that stochastic expression of CyaA may contribute to the heterogeneous levels of cAMP activity and RPs.

### (p)ppGpp/DksA represses RP expression in single cells

Previous work in *E. coli* shows that DksA and (p)ppGpp directly attenuate transcription of RP genes (*8*). A recent study also demonstrates that deleting *dksA* increases the rRNA levels in single *Salmonella* cells (*12*). We found that deleting *dksA* increased absorbance at 600 nm (*A*_600_)–normalized bL19-mGL and uS2-mGL levels in the stationary phase (22 hours) using a batch culture assay (fig. S13). In single cells, removing *dksA* also increased the mean fluorescence of bL19-mGl and uS2-mGL (Fig. 2). This increase is a shift of the distribution peak without affecting the high-RP tail, resulting in a decrease in their heterogeneity as measured by CV (Fig. 2). We also found that complementation of the *dksA* gene in Δ*dksA* cells restored the bL19-mGL heterogeneity to the WT level (fig. S9). Deleting *relA* and *spoT*, which abolished (p)ppGpp synthesis, had the same effects on bL19 variations as deleting *dksA* (fig. S11), supporting a coupled role of (p)ppGpp and DksA in RP regulation. The Δ*dksA*/*cyaA* double mutant displayed similar levels of mean RPs and heterogeneity as the Δ*dksA* single mutant (Fig. 2), indicating that the repression of RP expression by (p)ppGpp/DksA occurs downstream of cAMP/CRP.

### Heterogeneity of RP levels is caused by carbon starvation

Growth of *E. coli* in LB has been shown to increase medium pH in the stationary phase (*51*). We also noticed a slight increase in the medium pH of *Salmonella* cultures at 22 hours (fig. S14A) and thus tested the heterogeneity of bL19 in buffered LB (fig. S14). We observed that the CV of bL19-mGL significantly decreased upon deletion of *cyaA* or *dksA* in buffered LB (fig. S14, B and C), suggesting that the effects of cAMP/CRP and (p)ppGpp/DksA on RP variations do not depend on the pH increase.

Growth of *E. coli* in LB is limited by the carbon source in the stationary phase (*52*). Adding extra carbon sources (glucose, glycerol, mannose, casamino acids, or mannitol) in WT cells pregrown in LB for 5 hours significantly reduced the CV of bL19-mGL levels (fig. S15), supporting the notion that carbon starvation promotes RP variations.

### DksA enhances the promoter noise of *rpsB*

**Fig. 4.**
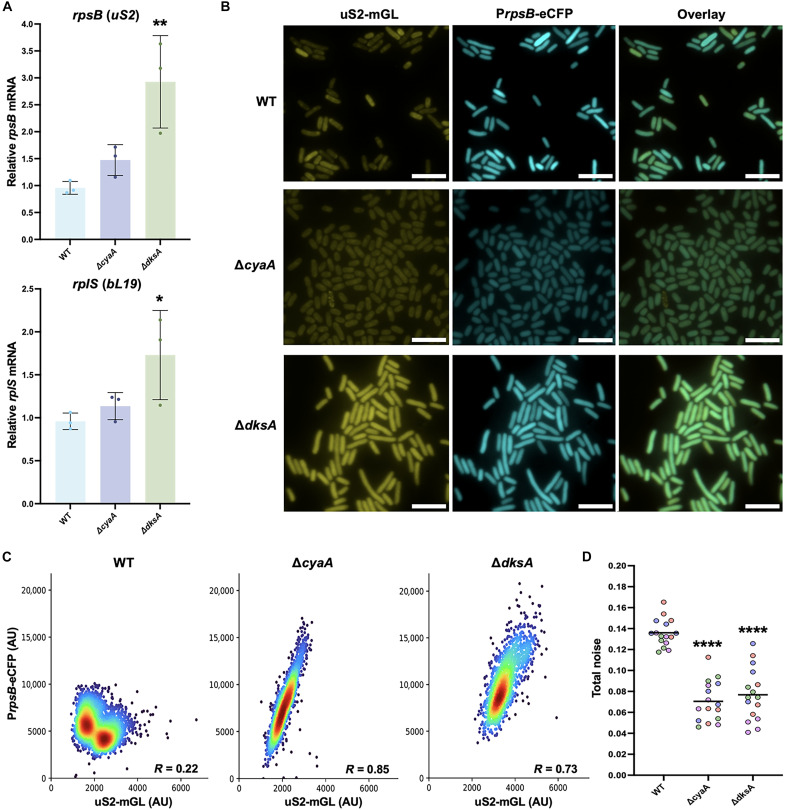
Effects of CyaA and DksA on *rpsB* promoter activity. (**A**) Relative mRNA levels determined by qRT-PCR. (**B**) Representative fluorescence microscopy images. Expression of P*rpsB-*eCFP is controlled by the promoter of uS2. Exposure time of the eCFP channel: WT (1/15 s), Δ*cyaA* (1/50 s), and Δ*cyaA* (1/25 s). (**C**) Scatterplots of P*rpsB-*eCFP versus uS2-mGL. (**D**) Total noise between the expression levels of eCFP and mGL in (C). The total noise was calculated as described (*19*). Biological replicates are indicated in different colors, and technical replicates are shown in the same colors. Error bars represent one standard deviation from the means. The *P* values are calculated using the one-way ANOVA with Dunnett’s test comparing mutants with the WT. *P < 0.05, **P < 0.01, and ****P < 0.0001. Scale bars, 5 μm.

We further investigated how CyaA and DksA affected the RP levels. Using the quantitative reverse-transcription polymerase chain reaction (qRT-PCR), we found that the mRNA levels of *rpsB* and *rplS* increased upon deletion of *dksA* but had little change when *cyaA* was deleted (Fig. 4A). We next constructed a promoter reporter of the *rpsB* operon. Deleting *dksA* increased the mean level of P*rpsB*-eCFP (Fig. 4, B and C). P*rpsB*-eCFP and uS2-mGL are under the control of the same promoter. Unexpectedly, the levels of P*rpsB*-eCFP and uS2-mGL were only weakly correlated among single cells (Fig. 4B), with a large total noise between the two (Fig. 4D). Deleting *dksA* significantly enhanced the correlation between P*rpsB-*eCFP and uS2-mGL (Fig. 4, C and D). Collectively, our results indicate that DksA represses *rpsB* transcription and contributes to the transcriptional noise at the *rpsB* promoter.

### cAMP promotes heterogeneity in cell growth and RP dilution

**Fig. 5.**
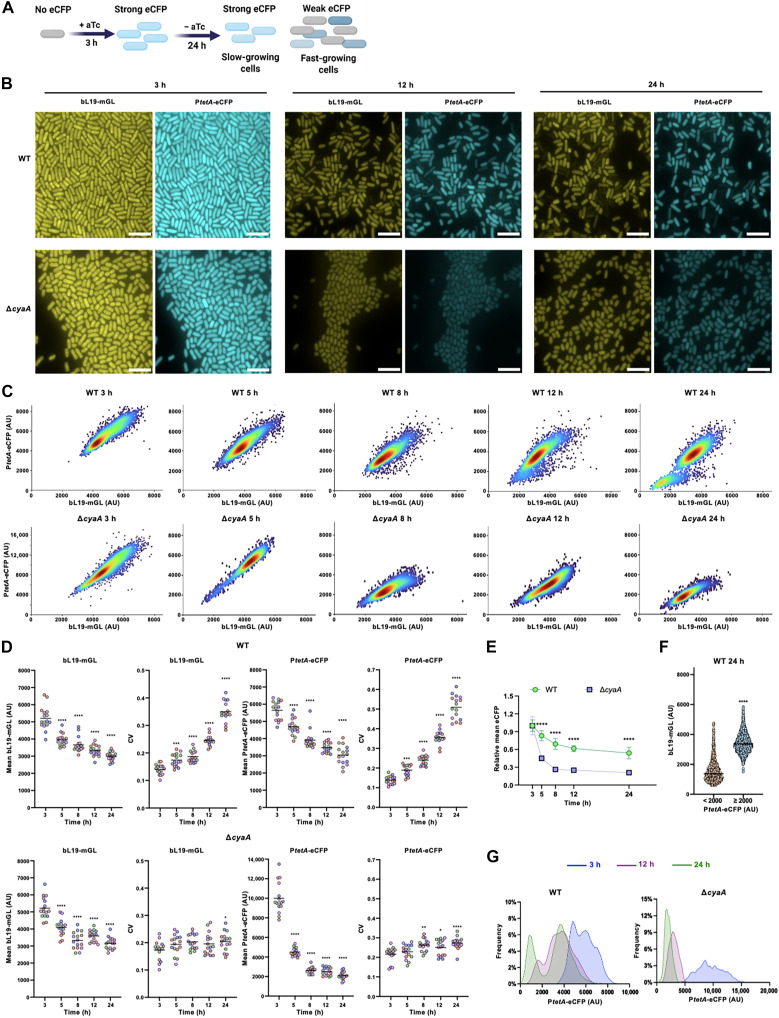
Heterogeneity in cell growth and ribosome dilution. (**A**) Schematic of the fluorescence dilution assay. Chromosomal P*tetA*-eCFP is induced with aTc (25 ng/ml) for 3 hours. The inducer aTc is then removed by medium exchange, and the cells are further cultured in 3-hour spent LB media. Upon removal of aTc, eCFP is not synthesized or degraded. Slow-growing cells maintain strong eCFP signals, whereas fast-growing cells have diluted eCFP fluorescence. (**B**) Representative fluorescence images. (**C**) Scatterplots of P*tetA-*eCFP versus bL19-mGL over time. (**D**) Changes in mean fluorescence and CV. Biological replicates are indicated in different colors, and technical replicates are shown in the same colors. (**E**) Time course of mean P*tetA*-eCFP. Error bars represent one standard deviation from the means. (**F**) Dot plot of WT cells after dilution, grouped by P*tetA-*ECFP levels. (**G**) Distribution of eCFP in (C). The images and analyses are representatives of at least three biological replicates. The *P* values are calculated using the one-way ANOVA with Dunnett’s test comparing mutants with the WT (D) or unpaired *t* test with Welch’s correction (E). *P < 0.05, **P < 0.01, ***P < 0.001, ***P < 0.001, and ****P < 0.0001. Scale bars, 5 μm.

To further understand the sources of variation in the cellular RP content, particularly to see how the RP content may be related to the extent of cell growth, we performed a fluorescence dilution assay (Fig. 5) (*53*). Briefly, we induced chromosomally incorporated P*tetA*-eCFP expression with aTc (*49*) at the time of inoculation into fresh LB (0 hours). After 3 hours of growth, the inducer was removed, and the cells were allowed to grow further in LB medium taken from an uninduced parallel culture (see Materials and Methods). Cells were collected at various time points later for microscopic analysis. Because eCFP was stable (figs. S2 and S16) and little eCFP was expressed after aTc removal at 3 hours (fig. S17), a decrease in the cellular eCFP signal would be attributed to dilution, indicating the growth and division of that cell. The possible relationship between RP content and the cellular growth status could then be revealed through an analysis of the eCFP and bL19-mGL signals.

Overall, both the mean levels of bL19-mGL and eCFP decreased over time (Fig. 5, D and E). However, the CVs of both bL19-mGL and eCFP increased over time (Fig. 5D). Intriguingly, a bifurcation of WT *Salmonella* cells started at around 12 hours (Fig. 5C), developing into two fully distinct subpopulations by 24 hours (9 and 21 hours after inducer removal, respectively), with low-eCFP cells exhibiting low bL19-mGL levels (Fig. 5F). This indicates strong differences in growth and dilution among these isogenic cells upon entering the stationary phase. A closer scrutiny of Fig. 5C shows a larger subpopulation of cells whose eCFP levels stopped changing from 8 hours onward and a smaller subpopulation of cells whose eCFP levels continued to decrease. As the decrease in eCFP level reflects continued cell growth in this experiment, the data suggest the existence of a minority subpopulation of cells that continued to grow past 8 hours, while the larger majority of cells stopped growing by 8 hours. Furthermore, the strong correlation between eCFP and bL19-mGL suggests that the minor subpopulation of cells with low bL19-mGL corresponds to those cells that continued to grow past 8 hours, and the major subpopulation with high bL19-mGL corresponds to those that stopped growing by 8 hours. In other words, those cells with high bL19-mGL resulted from their lack of dilution when growth stopped. In support of this notion, we found that stationary-phase cells with high bL19-mGL levels were larger (fig. S4, C and F) and more tolerant to ampicillin (fig. S18), a β-lactam antibiotic that targets the cell wall synthesis of growing cells.

Similar to WT cells, the same fluorescence dilution experiment applied to Δ*dksA* cells resulted in two subpopulations of fluorescence levels reflecting distinct growth histories of these cells, with a minority subpopulation that grew more than the majority (fig. S19). Notably, deleting *cyaA* abolished the distinct subpopulations (Fig. 5, C and G): eCFP fluorescence dropped more in Δ*cyaA* cells than in WT by 8 hours, but the dynamics were largely frozen afterward, indicating that Δ*cyaA* cells grew more than WT in the first 8 hours but stopped growing together afterward. At 24 hours, the distribution of the fluorescence levels of bL19-mGL and eCFP for Δ*cyaA* cells was centered between the two peaks of WT cells (Fig. 5, C and G). Together, these data suggest that it was cAMP signaling that enabled the heterogeneous growth of WT cells, with those not growing after 8 hours showing higher ribosome content and those that continued growing to some extent after 8 hours showing lower ribosome content, most likely by experiencing further dilution because of growth. We will show below that the different growth behaviors inferred from the results of the fluorescence dilution assay are well supported by a more quantitative examination of the growth curves.

### Heterogeneity in gene expression induced in the stationary phase

**Fig. 6.**
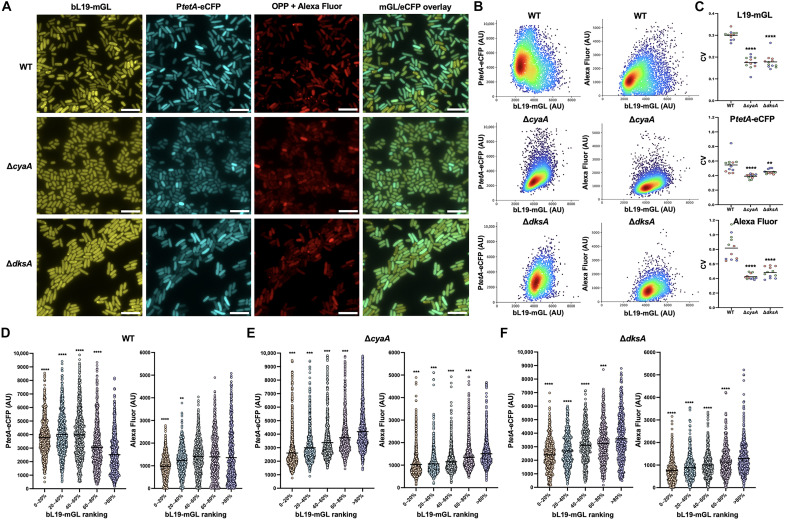
Gene expression in stationary-phase cells. (**A**) Cells carrying pZS-P*tet*-eCFP were grown to 19 hours and induced with aTc (250 ng/ml) and 20 μM OPP for 3 hours. (**B**) Scatterplots of fluorescence signals quantified from (A). (**C**) CV of fluorescence signals. Biological replicates are indicated in different colors, and technical replicates are shown in the same colors. (**D** to **F**) Bin analyses of fluorescence signals based on the ranking of bL19-mGL levels from low to high. Expression of P*tetA*-eCFP decreased in the subpopulation with the highest bL19 level. The *P* values are calculated using the one-way ANOVA with Dunnett’s test compared with the >80% subpopulation. The images and bin analyses are representatives of at least three biological replicates. **P < 0.01, ***P < 0.001, and ****P < 0.0001. Scale bars, 5 μm.

Protein synthesis in the stationary phase is generally not well understood. Heterogeneity in stationary-phase gene expression has been widely reported (*7*, *21*, *54*), but the cause is not well understood. We used the pZS P*tetA-*eCFP plasmid to characterize the relationship between inducible gene expression and the cellular RP level. The expression of P*tetA-*eCFP was induced with aTc in the stationary phase (at 19 hours) in bL19-labeled cells, and single-cell eCFP fluorescence was characterized 3 hours later (at 22 hours) (Fig. 6). We also characterized the overall extent of protein synthesis during those 3 hours with *O-*propargyl-puromycin (OPP) (*55*). The amount of aTc and OPP used did not affect cell growth or protein production (fig. S20). We observed a general anticorrelation between eCFP and bL19-mGL levels for WT cells (Fig. 6, A and B), with those cells with the top 20% highest bL19 levels exhibiting the lowest expression of *PtetA-*eCFP (Fig. 6D). A chromosomal P*tet*-eCFP reporter revealed a similar trend (fig. S21). This trend was not dependent on ribosome hibernation (fig. S22). Given that the fluorescence dilution experiments above established a general negative correlation between the bL19 levels and the cellular growth status after ~8 hours (Fig. 5), the anticorrelation between eCFP and bL19 levels indicates that it was mostly the growing subpopulation (those with low bL19 levels) that responded to the induction of eCFP expression, while the subpopulation of nongrowing cells responded weakly to induction. This conclusion is consistent with the molecular knowledge that cells entering the stationary phase rapidly inhibit transcription driven by the housekeeping σ factor σ^70^ (*56*). In contrast, the overall protein synthesis displayed the opposite trend (Fig. 6, B and D), indicating that the ribosomes remained active in the nongrowing cells, presumably engaged in synthesizing stress proteins such as those driven by the general stress σ factor σ^S^ (RpoS) (*33*, *34*). To further test this, we analyzed inducible gene expression and overall protein synthesis in the Δ*rpoS* mutant. As expected, deleting *rpoS* did not change the pattern that high-RP cells exhibited lower inducible expression of P*tetA*-eCFP because P*tetA* is not regulated by RpoS (fig. S23). In contrast to the WT strain (Fig. 6), the overall protein synthesis in the Δ*rpoS* strain, measured by OPP incorporation, was lower in high-RP cells (fig. S23), supporting our conclusion that ribosomes in the high-RP cells are heavily involved in translating RpoS-dependent genes.

Given that cAMP/CRP and (p)ppGpp/DksA promote variations in cellular RP content (Fig. 2 and figs. S9 to S11), we further tested how these factors might affect the heterogeneity in inducible gene expression for mutant strains in the stationary phase. The negative correlation between RP levels and inducible gene expression was no longer observed in the Δ*cyaA* and Δ*dksA* mutants (Fig. 6, E and F). Furthermore, deleting *cyaA* or *dksA* significantly reduced the heterogeneity in P*tetA*-eCFP and total protein synthesis (Fig. 6C), indicating that cAMP/CRP and (p)ppGpp/DksA also enabled cell-to-cell variations in gene expression during the stationary phase.

### Heterogeneity in the accumulation of *Salmonella* virulence gene products

**Fig. 7.**
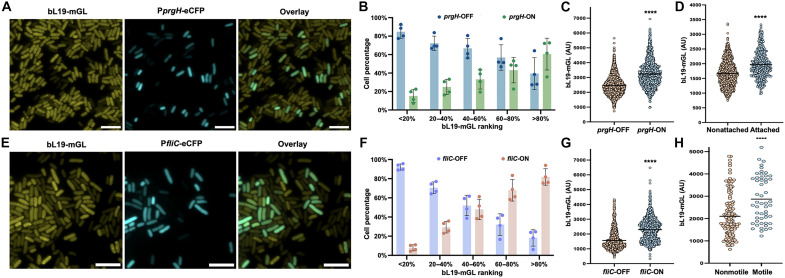
Expression of virulence genes with fluctuating ribosome content. (**A** and **E**) Fluorescence microscopy images of *Salmonella* cells at 22 hours. P*prgH*-eCFP and P*fliC*-eCFP indicate the expression of SPI-1 and flagellar pathways, respectively. (**B** and **F**) Bin analyses based on the ranking of bL19-mGL levels from low to high. Error bars represent one standard deviation from the means. (**C** and **G**) Scatterplots of fluorescence signals quantified from (A) and (E). (**D**) Distribution of bL19-mGL fluorescence in WT cells attached or not attached to HeLa cells. (**H**) Distribution of bL19-mGL fluorescence in motile and nonmotile cells. The images are representatives of at least three biological replicates. The *P* values are calculated using the unpaired *t* test with Welch’s correction. ****P < 0.0001. Scale bars, 5 μm.

We further analyzed heterogeneity in gene expression from two key virulence pathways, SPI-1 and flagella. SPI-1 genes are critical for *Salmonella* to invade host cells, while the flagella enable *Salmonella* cells to move within the host (*57*–*60*). The expressions of SPI-1 and flagellar genes are known to be bistable in the late exponential phase (e.g., at 4 to 5 hours), with only a subpopulation of *Salmonella* cells expressing these genes (*61*, *62*). To test possible relations between the heterogeneous expression of the virulence genes with heterogeneity in cellular growth phases, we constructed fluorescent reporters driven by the promoters of selected virulence genes, P*prgH*-eCFP (SPI-1 reporter) and P*fliC*-eCFP (flagellar reporter), and expressed them from a low-copy-number plasmid (pZS) in bL19-mGL cells (Fig. 7). At the batch culture level, we found that the flagellar promoter mainly synthesized eCFP during growth (before ~6 hours; fig. S24), while the SPI-1 promoter continued synthesizing eCFP after growth slowed down (until ~10 hours; fig. S24).

At the single-cell level, cells with high bL19 levels (corresponding to those not growing after 8 hours; see Fig. 6) showed higher accumulation of eCFP from both the promoters of SPI-1 and flagella (Fig. 7). Together, the batch culture and single-cell data suggest that the products of virulence genes are accumulated mostly during exponential growth; the heterogeneity in cellular abundance largely reflects differences in dilution, with the subpopulation of cells that experienced sudden growth arrest showing increased levels of eCFP and bL19-mG fluorescence. Expectedly, deleting *cyaA*, which removed the subpopulation showing sudden growth arrest (Fig. 5B), coincided with significantly reduced fractions of *prgH*-ON and *fliC-*ON cells (fig. S25).

To test whether cells with various RP levels behave differently, we performed HeLa cell attachment and single-cell motility assays. *Salmonella* cells attached to HeLa cells had significantly higher levels of bL19-mGL than those in the supernatant (Fig. 7D), and motile cells also displayed higher bL19-mGL fluorescence than nonmotile cells (Fig. 7H). These results were consistent with the higher expression of SPI-1 and flagellar genes in high-RP cells.

### Protein expression during the exponential and stationary phases

**Fig. 8.**
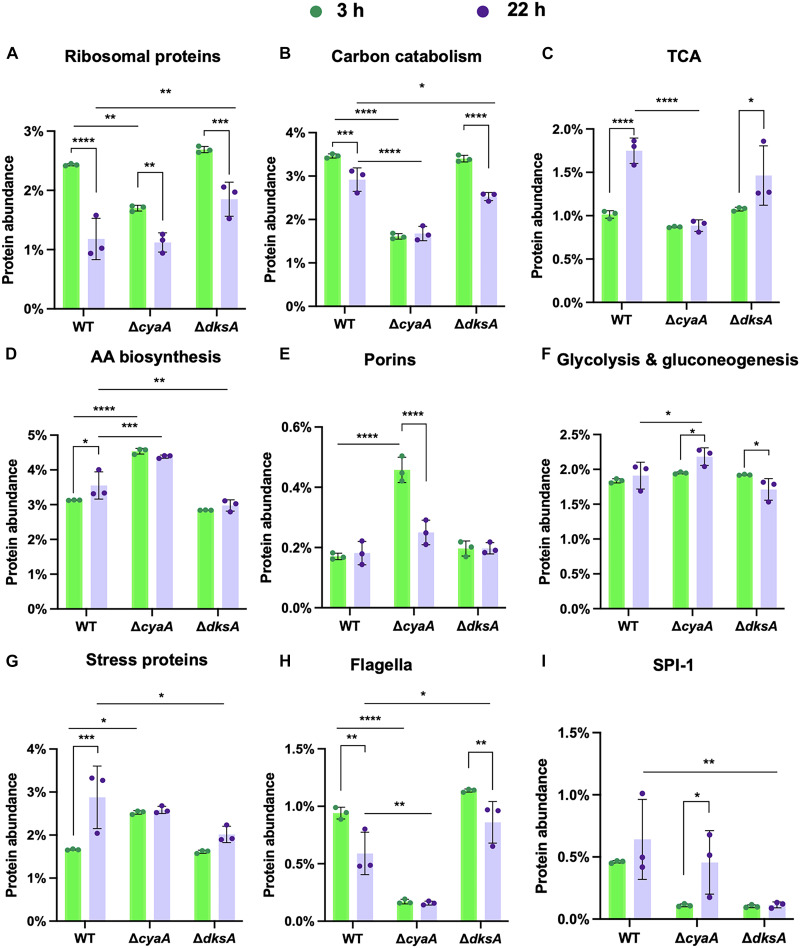
Proteomics analyses of *Salmonella* strains grown in LB at 37°C. Relative abundance of proteins in the following pathways: (**A**) RPs, (**B**) carbon catabolism, (**C**) TCA cycle, (**D**) amino acid (AA) biosynthesis, (**E**) porins, (**F**) glycolysis and gluconeogenesis, (**G**) stress proteins, (**H**) flagella, and (**I**) SPI-1. Protein abundances for selected protein groups were obtained by summing the protein abundances of all members of the protein group as defined in table S3 and listed in table S4. Error bars represent one standard deviation from the means. The *P* values are calculated using the two-way ANOVA with Tukey’s post hoc test. *P < 0.05, **P < 0.01, ***P < 0.001, and ****P < 0.0001.

To test more broadly how protein expression was affected by growth phases, cAMP/CRP, and (p)ppGpp/DksA, we performed proteomic analysis of WT, Δ*cyaA*, and Δ*dksA* cells harvested during the exponential (3 hours) and stationary (22 hours) phases using tandem mass tag (TMT) isobaric labeling (*63*). Although known to be affected by ratio compression effects (*64*), TMT allows for deep protein coverage (3000+ proteins detected in each of our samples). Raw peptide data were converted to protein abundances and reported as fractions of total protein mass using the xTop methodology (see Materials and Methods, with data listed in table S3) (*65*, *66*). We then added up the abundances of all proteins belonging to various functional groups (defined in table S3). The results on the total abundance of each functional group, listed in table S4, are plotted in Fig. 8 for several representative groups for the six combinations of strains and growth phases characterized.

We first examined the abundances of RPs (Fig. 8A). Overall, the RP levels drop for the culture at 22 hours compared to 3 hours, consistent with a decrease in the mean fluorescence of bL19-mGL and uS2-mGL in Fig. 1D. The similarity of RP levels between WT and Δ*cyaA* cells and the increase in RPs for Δ*dksA* cells in the stationary phase (22 hours) are in good agreement with the mean bL19-mG and uS2-mG fluorescence levels (Fig. 2B). Also, the similarity of the abundance ratios across the individual RPs (fig. S26) affirms the consistency of the relative quantification in our data. However, the absolute quantitation is clearly affected by the ratio compression phenomenon, as the levels of RPs are known to be around 40% for *E. coli* cells growing in rich media (*66*) and similarly for *Bacillus subtilis* and *Vibrio natriegens* (*67*), more than 10× larger than that found here. Thus, we take TMT proteomics to be reliable primarily at the relative level. However, we also report below the absolute abundance across proteins, as it does provide some information on the order of magnitude of the abundances of different protein classes.

We next investigated the differences across the various strains. For RPs, a clear difference is seen between WT and Δ*cyaA* cells at 3 hours (Fig. 8A), suggesting that Δ*cyaA* cells grew more slowly during the exponential phase. The effect of *cyaA* deletion is clearly seen in the reduced levels of enzymes of carbon catabolism of the tricarboxylic acid (TCA) cycle at 3 and 22 hours (Fig. 8, B and C). For WT and Δ*dksA* cells, the rise of TCA cycle enzymes at 22 hours compared to 3 hours (Fig. 8C) is consistent with the known up-regulation of cAMP-CRP under carbon limitation (*41*). The small change for carbon catabolic enzymes in WT and Δ*dksA* cells (Fig. 8B) is somewhat puzzling at first sight. A closer examination shows that it is a result of a big drop in the level of MalE (fig. S27A) along with the up-regulation of other carbon catabolic enzymes (fig. S27, B and C, and table S3) from 3 to 22 hours, presumably reflecting a depletion of maltose in LB and switching to other C-sources in the medium, e.g., acetate, which is heavily excreted during exponential growth in LB (*68*). Δ*cyaA* cells show increased synthesis of biosynthetic enzymes during exponential growth compared to WT cells (Fig. 8D), consistent with their misallocation of the proteome reported previously (*38*, *69*). They up-regulate porin expression (Fig. 8E), possibly as a compensation for their muted response in carbon catabolism (Fig. 8B). However, there is little adjustment in other components of central carbon metabolism (Fig. 8F).

A very noteworthy change in the expression pattern of Δ*cyaA* cells is the up-regulation of stress proteins already during exponential growth to levels comparable to what WT cells would reach later in the stationary phase (Fig. 8G). Looking into individual stress proteins, we see that RpoS, the general stress response σ factor itself, is already high at 3 hours for Δ*cyaA* cells, reaching a level that WT cells reach only after strong up-regulation at 22 hours (fig. S27D). Expectedly, this difference in RpoS protein level is reflected in the expression levels of members of the RpoS regulon (see fig. S27, E and F, and table S3) (*33*). Last, we report the expression profiles for the *Salmonella* virulence genes: The flagellar pathway is strongly down-regulated in Δ*cyaA* cells (Fig. 8H), consistent with cAMP-CRP–dependent up-regulation of flagellar synthesis in *Salmonella* (*70*). The expression of SPI pathway proteins is also affected by cAMP during exponential growth (Fig. 8I), which is in line with previous reports (*71*, *72*).

### General feature of cAMP signaling in RP variations under various starvation conditions

**Fig. 9.**
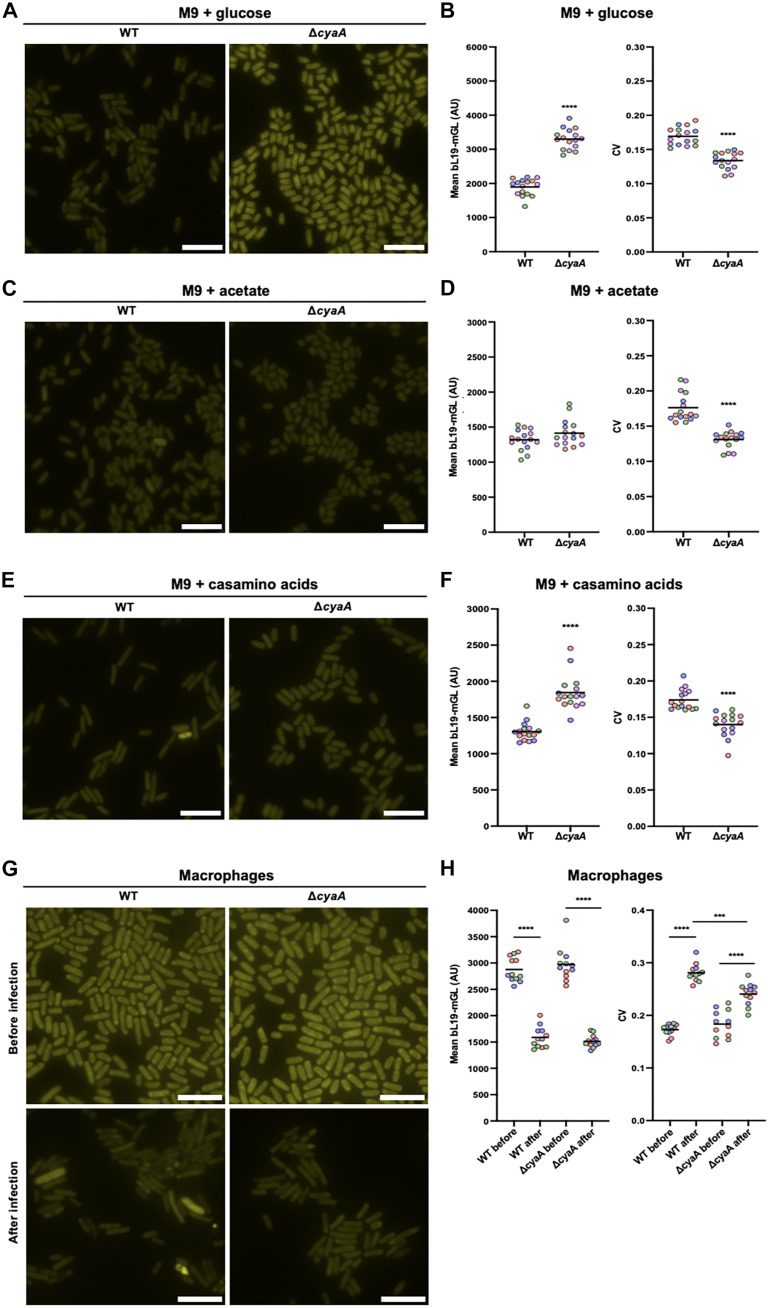
Heterogeneity of RP levels under various growth conditions. WT and Δ*cyaA* bL19-mGL cells were grown in minimal media (M9) with various carbon sources or inside macrophages for 22 hours. (**A**, **C**, **E**, and **G**) Representative fluorescence microscopy images of *Salmonella* cells. (**B**, **D**, **F**, and **H**) Means and heterogeneity of bL19 fluorescence in single cells. Biological replicates are indicated in different colors, and technical replicates are shown in the same colors. The *P* values are calculated using the two-way ANOVA with Tukey’s post hoc test (D) or unpaired *t* tests with Welch’s correction. ***P < 0.001 and ****P < 0.0001. Scale bars, 5 μm.

cAMP plays a key role in carbon metabolism (*37*, *38*). The main carbon source in LB is catabolizable amino acids (*52*). To test whether cAMP affects RP variations in other carbon sources, we tested bL19-mGL levels in stationary-phase cells grown in minimal media M9 with glucose, acetate, or casamino acids as the sole carbon source. We found that deleting *cyaA* decreased the CV of bL19-mGL in all tested medium conditions (Fig. 9, A to F). In addition, switching cells from M9 + glucose or M9 + casamino acids to M9 salts without any carbon source increased the CV of bL19-mGL, which was reduced in the Δ*cyaA* cells (fig. S28). To further test complex carbon sources in natural environments, we allowed WT and Δ*cyaA Salmonella* cells to grow inside J774A.1 macrophage cells. WT cells grown in macrophages displayed large heterogeneity in bL19-mGL levels, whereas such heterogeneity was significantly decreased in the Δ*cyaA* cells (Fig. 9, G and H). Together, our results suggest that cAMP plays a general role in promoting RP variations during starvation.

## DISCUSSION

Protein synthesis is a resource-intensive process, and adjusting the ribosome level is critical for cell growth and adaptation to the environment. Previous studies have established that the growth rate of the cell population positively correlates with the abundance of ribosomes during exponential growth (*6*). In general, little is known about protein synthesis in the stationary phase despite the fact that the stationary phase is a pervasive cellular state of bacteria and exhibits distinct characteristics compared to the exponential growth phase. Stationary-phase cells are smaller and less metabolically active than rapidly growing cells but are generally more tolerant to stresses and antimicrobials (*7*, *16*, *33*, *73*). Stationary-phase cells are phenotypically diverse (*7*). A recent study demonstrates that in *B. subtilis*, a fraction of stationary-phase cells is able to synthesize proteins, and removing (p)ppGpp increases translation in individual cells (*55*). Our knowledge of protein synthesis during the stationary phase is even less at the single-cell level. Here, we simultaneously characterized the levels of ribosomes and the expression of inducible and virulence genes for individual *Salmonella* cells during the transition to the stationary phase for WT cells and two mutants lacking signaling pathways important to this transition, Δ*cyaA* and Δ*dksA*. In addition, we characterized the proteome of these strains during exponential growth (3 hours) and in the stationary phase (22 hours).

A plethora of results are generated from this study. First, WT cells exhibit strong heterogeneity in RP levels upon entering stationarity (Fig. 1). This heterogeneity is decreased in cells deleted of *cyaA* and *dksA*, with the reduced heterogeneity of Δ*cyaA* cells coming without changes in the mean RP levels (Fig. 2). The heterogeneity in the RP levels of WT cells is coupled to the heterogeneity in gene expression: We find that the expression of genes induced after entering the stationary phase is negatively correlated with RP levels (Fig. 6), while the accumulation of virulence gene products during the transition is positively correlated with RP levels (Fig. 7). These intriguing findings can all be rationalized in terms of differential dilution of the expressed gene products by cells that grow differently during the transition: As revealed by a fluorescence dilution assay, those WT cells with higher RP levels diluted less, while those with lower RP levels diluted more (Fig. 5). The results lead to a model on the differential growth of WT cells as nutrients are depleted, with a fraction of cells abruptly stopping growth and another fraction gradually slowing down growth as nutrients are depleted. Because RPs are stable (fig. S2), the cells with abrupt growth arrest would then end up having higher RP levels because of the lack of dilution, while the other cells would have lower RP levels because of dilution. This differential dilution model accounts for the correlation of the product of virulence gene expression with RP levels (Fig. 7), given that both are accumulated during growth, and their levels reflect the degree of dilution as nutrients are depleted. This model also accounts for the anticorrelation of the product of genes induced after entering the stationary phase (Fig. 6), as the cells that gradually slow down growth can still synthesize genes when induced in the late hours, while the growth-arrested cells are known to strongly reduce gene expression (*56*).

Because Δ*cyaA* cells exhibited much reduced heterogeneity, with RP levels and gene expression resembling the (minor) subpopulation of WT cells with low RP levels, hence more dilution (Figs. 4 to 6), we can infer that this subpopulation of WT cells had its cAMP signaling muted, and it was the other (major) subpopulation of cells showing abrupt growth arrest that had cAMP signaling active. In other words, cAMP signaling guided this major subpopulation to continue growing during the course of nutrient depletion, only to crash with abrupt growth arrest when nutrients were truly depleted. The scenarios depicted here for WT and Δ*cyaA* cells are supported by the results of proteomic analysis (Fig. 8), which shows that the proteome of WT cells is configured for growth early on and then makes a transition to the stationary phase (increasing stress proteins and TCA enzymes for energy while dialing down RPs) only as the stationary phase is entered. In contrast, the exponential-phase proteome of Δ*cyaA* cells is already configured for slow growth and is similar to the stationary phase (lower RPs and increased stress proteins).

The differences in proteome allocation are corroborated by features exhibited by the growth curves: A closer, quantitative examination of the growth curves (figs. S29 and S30) shows that WT cells exhibit several distinct regimes during the course of nutrient depletion (red circles in fig. S29), with an exponential growth regime in the first 2 to 3 hours, followed by an intermediate regime of slower growth (5 to 8 hours) and an abrupt slowdown of growth at ~8 hours. In contrast, the growth curve of Δ*cyaA* cells (blue triangles in fig. S29) does not exhibit the intermediate regime. While their exponential growth is slower than WT cells (consistent with their lower RP levels at 3 hours), this growth phase lasts longer (~5 hours) and transitions directly to the same barely growing state as WT cells at ~8 hours. However, because of their more extended growth regime, they accumulate more biomass (higher optical density) and hence experience more dilution, supporting the scenarios depicted in the differential dilution model. Note that such quantitative interpretation of the growth curves requires linearity between optical density and biomass, which is established through quantitative calibration (fig. S30).

The differential dilution model posits that WT cells are composed of two subpopulations of cells as nutrients are depleted, with one subpopulation being similar to Δ*cyaA* cells, keeping a slower but more expanded growth regime accompanied by lower levels of RPs and higher levels of stress proteins. The other subpopulation attempts to keep growing by adapting its proteome to whatever nutrients are left in the medium (e.g., expressing acetate utilization to switch to growth on acetate; see fig. S27B). However, because of the high nutrient requirement (and, presumably, the depletion of oxygen) for cultures that reach very high cell density in the late hours (fig. S29), these cells cannot maintain growth for long, even with cAMP-guided adaptation, and end up experiencing abrupt growth arrest accompanied by higher RP levels because of the lack of dilution. Thus, in this scenario, the cells get “tricked” into abrupt growth arrest by the temporary relief of the initial nutrient depletion (say, around 5 hours in fig. S29) because of proteome adaptation enabled by cAMP signaling. This may reflect a regulatory program that is intended to give the cells a competitive edge as nutrients deplete in a complex community but comes across as overzealous in the monoculture laboratory condition, or it may be a way to give the stationary-phase cells more ribosomes, which would enable them to recover more rapidly when nutrients return. Now, the mechanism by which CyaA enables this heterogeneous response to starvation by WT cells is unknown. It could be a passive consequence of heterogeneity in the level of CyaA [which is known to be a low-abundant protein (*74*)]. Alternatively, it could be a result of active regulation that drives a bistable response to adaptation as nutrients are depleted because of a hitherto unknown regulatory circuit. The connection between the cAMP/CRP and (p)ppGpp/DksA pathways on RP levels also remains unclear at this point and warrants future studies. We found that the level and heterogeneity of bL19 in the Δ*dksA*/Δ*cyaA* double mutant were similar to those in Δ*dksA* (Fig. 2), suggesting that the (p)ppGpp/DksA pathway likely plays a role downstream of cAMP/CRP. Our mass spectrometry (MS) result revealed that amino acid biosynthesis proteins, which are up-regulated by (p)ppGpp/DksA (*9*, *10*), were increased in Δ*cyaA* (Fig. 8D), indicating that removing cAMP activates the ppGpp/DksA pathway. A plausible model is that cAMP represses (p)ppGpp/DksA activity in some cells to cause poor adaptation to starvation and slower growth, leading to higher ribosome levels in those cells. Future work using single-cell (p)ppGpp reporters would help test this model. Regardless, what our study reveals here is an unexpected role of cAMP signaling in promoting ribosome variations and gene expression heterogeneity among single cells through differential growth and dilution as they enter the stationary phase.

## MATERIALS AND METHODS

### Bacterial strains, plasmids, and culture conditions

All bacterial strains and key plasmids used in this study are detailed in the key resources table (table S1). The oligonucleotides used for the construction of mutant strains and plasmids are provided in table S2. All *Salmonella* strains were derived from *S. enterica* serovar Typhimurium ATCC (American Type Culture Collection) 14028s, and the *E. coli* strain used in this study was K-12 MG1655. Bacterial cells kept in glycerol stocks at −80°C were streaked onto LB agar plates and incubated overnight at 37°C. A single colony was picked and inoculated into LB Lennox medium [NaCl (5 g/liter), tryptone (10 g/liter), and yeast extract (5 g/liter)] to generate seed cultures. Seed cultures were subsequently transferred into LB medium and grown at 37°C with aeration for 3, 5, or 22 hours. Where required, antibiotics were added at the following concentrations: ampicillin (100 μg/ml), spectinomycin (300 μg/ml), and chloramphenicol (1 or 25 μg/ml). For induction of P*tetA*- or P*araBAD*-controlled fluorescent protein reporters, l-arabinose and aTc were used at final concentrations of 1 mM and 250 ng/ml, respectively, unless otherwise specified. The M9 medium contains Na_2_HPO_4_ (6.78 g/liter), KH_2_PO_4_ (3 g/liter), NH_4_Cl (1 g/liter), NaCl (0.5 g/liter), and specified carbon sources.

To visualize the ribosomes, we used a construction similar to the lambda-Red recombination (*75*) where the stop codon of *rpsB* or *rplS* was replaced by *mGL*::*FRT*::*cat* (chloramphenicol acetyl transferase)::*FRT* or *eCFP*::*FRT*::*cat*::*FRT* via a flexible linker 2XGGGGS. To construct the strain bL19-mGL-P*tetA*-eCFP to monitor cell growth, the P*tetA*-*eCFP-cat* cassette was amplified from the plasmids pZS-P*tetA*-*eCFP-cat* by PCR. The cassette was integrated into the chromosomal region between genomic coordinates 3114907 and 3115482 through Red recombinase–mediated recombination. The *cat* marker was subsequently removed using the helper plasmid pCP20 (*75*).

All in-frame gene deletion mutants were generated by lambda-Red recombination (*75*) using the *FRT*::*cat*::*FRT* cassette amplified from pKD3, with homology regions flanking the target gene. The recombinant cells were selected on LB agar plates containing chloramphenicol, and successful deletions were confirmed by PCR.

To construct the pZS-P*tet*-mCherry-P*rpsB*-eCFP plasmid, the promoter region of the *rpsB* gene was amplified from the *S. enterica* serovar Typhimurium ATCC 14028s chromosome and cloned into pZS-P*tet*-mCherry-P*mgtA*-eCFP by restriction cloning using the BstB I and Sal I sites. Complementation plasmids were generated by cloning the *cyaA* and *dksA* genes, including their native promoters, into pZS-P*tet-*eCFP at the Xho I and Hind III restriction sites. The pZS plasmid contains the C101* origin and three or four copies per cell (*76*). The P*tetA* (p5205) and P*araBAD* (p5426) expression plasmids were gifts from M. Hensel (Universität Osnabrück, Osnabrück, Germany) (*49*). The promoter regions of P*tetA* and P*araBAD* were ligated into the plasmids pZS-P*tet*-eCFP or p*ZS*-P*tet*-mCherry through Gibson assembly.

### Growth and fluorescence determination by a plate reader

Overnight cultures were diluted 1:50 in fresh LB and distributed evenly into a 96-well microplate. Growth and fluorescence intensity were measured every 20 min using a plate reader (Synergy HTX, BioTek) at 37°C for 24 hours with shaking. *A*_600_ was measured, and the optimal excitation (Ex) and emission (Em) wavelengths for each fluorescence protein were used as follows: mCherry: Ex = 575 nm and Em = 620 nm; GFP (green fluorescent protein): Ex = 485 nm and Em = 528 nm; eCFP: Ex = 420 nm and Em = 475 nm.

### Western blot analysis

Overnight bacterial cultures were diluted 1:50 in LB and grown at 37°C with shaking for an additional 3 or 22 hours. One milliliter of each culture was centrifuged, and the pellets were washed once with phosphate-buffered saline (PBS) and resuspended in 300 μl of PBS. The cells were lysed by sonication, and total proteins were quantified by the bicinchoninic acid assay, separated on 10% SDS–polyacrylamide gel electrophoresis, transferred onto a polyvinylidene fluoride membrane via wet transfer, and blocked in 5% nonfat milk in PBS with 0.2% Tween 20. For the detection of bL19-mGL and uS2-mGL proteins, the blocked membranes were probed by an anti-GFP primary antibody (diluted 1:1000). A primary antibody was detected using horseradish peroxidase–conjugated goat anti-mouse immunoglobulin G (diluted 1:10,000), followed by detection with enhanced chemiluminescence reagents and imaging on ChemiDoc Touch (Bio-Rad). Ponceau Red staining of the membrane was performed as a loading control. Images were cropped and analyzed using ImageJ/Fiji (*77*, *78*).

### Imaging

Overnight cultures were diluted 1:50 in LB and grown aerobically in 96-well plates at 37°C for 3, 5, and 22 hours before imaging. Cells were collected and concentrated into a final volume of ~10 μl, and 1 μl of concentrated culture was immobilized on 1.5% low-melting-point agarose containing LB medium. Fluorescence images were acquired using a BZ-X800 fluorescence microscope (Keyence) equipped with a Plan Apo ×100/1.45–numerical aperture oil-immersion objective. Excitation was provided by the built-in high-intensity light-emitting diode light source, in combination with the following filter sets: Cyan (Ex = 405/20 nm, Em = 460/50 nm), yellow fluorescent protein (Ex = 500/20 nm, Em = 535/30 nm), and Texas Red (Ex = 545/25 nm, Em = 605/70 nm). Images were processed and cropped using Fiji, and cells were segmented using Cellpose (*79*, *80*). For a direct comparison, all images displayed side by side were acquired, analyzed, and adjusted using identical settings. In a typical frame, more than 400 individual cells were analyzed. The fluorescence signal of individual cells represents the mean fluorescence intensity per pixel.

For superresolution imaging, cells were prepared as described above. Concentrated cultures were immobilized on 1.5% low-melting-point agarose containing LB medium and mounted on 10-well microscope slides. Agarose-mounted samples were imaged on a Zeiss LSM 980 Airyscan 2 confocal microscope equipped with a 63× oil lens for enhanced resolution and sensitivity. Fluorescence signals were excited with the appropriate laser lines and detected using spectral detectors optimized for each fluorophore.

### OPP labeling

Protein synthesis was assessed using the Click-iT Plus OPP Protein Synthesis Assay Kit (Invitrogen) according to the manufacturer’s instructions. Briefly, cells were grown in LB aerobically at 37°C. After 19 hours of growth, 3 μl of 2 mM OPP dissolved in dimethyl sulfoxide was added to 300 μl of the cultures. Cultures were returned to the 37°C shaker, and OPP incorporation was allowed to proceed for 3 hours. All subsequent steps were performed at room temperature. Cells were harvested by centrifugation at 5000*g* for 5 min and resuspended in 500 μl of 4% paraformaldehyde in PBS for fixation (15 min). Following fixation, cells were pelleted and permeabilized by resuspension in 500 μl of 70% ethanol for another 15 min. The cells were then incubated with 100 μl of 1× Click-iT Plus OPP reaction cocktail for 30 min in the dark to label OPP with Alexa Fluor. Last, cells were washed once with Click-iT Reaction Rinse Buffer and resuspended in 20 μl of PBS for microscopy.

### RNA extraction and qRT-PCR

To prepare total RNA for qRT-PCR analysis, bacterial cultures were grown in LB medium at 37°C with shaking until an *A*_600_ of ~1.5 was reached. One milliliter of each culture was harvested by centrifugation at 5000*g* for 10 min at 4°C. Cell pellets were immediately flash-frozen in liquid nitrogen and stored at −80°C until RNA extraction. Total RNA samples were isolated using the hot phenol method as previously described (*81*), and residual chromosomal DNA was removed by treatment with ribonuclease-free deoxyribonuclease I (NEB) according to the manufacturer’s instructions. For quantitative RT-PCR, up to 1 mg of DNA-free RNA was subjected to cDNA synthesis using the iScript cDNA Synthesis Kit (Bio-Rad). Real-time PCR was performed on a StepOnePlus Real-Time PCR System (Applied Biosystems) (*82*). The *rpoD* gene was used as the endogenous normalization control. Relative gene expression levels were calculated using the 2^−ΔΔCt^ method (*83*).

### Proteomics

Proteomics experiments were performed in triplicate. Overnight *Salmonella* cultures were diluted 1:50 in LB medium and grown at 37°C with shaking for either 3 or 22 hours. At given time points, 3 ml of culture was harvested, washed twice with PBS, and collected by centrifugation at 5000*g* for 5 min at room temperature. The resulting cell pellets were immediately flash-frozen in liquid nitrogen and stored at −80°C until further processing. For protein extraction, frozen cell pellets were resuspended in 400 μl of lysis buffer containing 100 mM Hepes (pH 8.0), 8 M urea, 0.5% SDS, and a 1× protease inhibitor cocktail (Sigma-Aldrich). Cells were lysed by sonication on ice using two cycles of 10-s pulses with 30-s intervals to minimize overheating. Total proteins were quantified by the bicinchoninic acid assay following the manufacturer’s instructions. Prepared protein samples were submitted for quantitative MS analysis at the Thermo Fisher Center for Multiplexed Proteomics at Harvard Medical School.

For 18-plex TMT proteomics, 25 μg of proteins from each sample was reduced with tris(2-carboxyethyl)phosphine, alkylated with iodoacetamide, and then further reduced with dithiothreitol. Proteins were precipitated onto SP3 beads to facilitate a buffer exchange into digestion buffer. Samples were digested with Lys-C (1:50) overnight at room temperature and trypsin (1:50) for 6 hours at 37°C. Peptides were labeled with TMTPro 18-plex reagents. Two microliters of each sample was pooled and used to shoot a ratio check to confirm complete TMT labeling and to allow for normalization of each sample. All 18 TMTPro-labeled samples were pooled according to the ratios determined from the ratio check. Peptides were desalted using a Sep-Pak, fractionated into 24 fractions using basic reverse-phase high-performance liquid chromatography. Twelve fractions were solubilized, desalted by a stage tip, and analyzed on an Orbitrap Eclipse.

MS2 spectra were searched using the COMET algorithm against a *S. enterica* Typhimurium UniProt composite database containing its reversed complement and known contaminants. Peptide spectral matches were filtered to a 1% false discovery rate using the target-decoy strategy combined with linear discriminant analysis. The proteins were filtered to a <1% false discovery rate and quantified only from peptides with a summed signal-to-noise threshold of >180 (18-Plex).

Protein abundances were estimated from the peptide spectra using xTop version 2.2 (*65*, *66*), available online at https://gitlab.com/mm87/xtop. The protein abundances were then multiplied by the protein molecular weights and normalized to unity to obtain the protein mass fractions reported in table S3. These protein mass fractions are summed for each functional group, and the total abundance of the group is reported in table S4.

### eCFP dilution assay

To monitor cell growth, a chromosomally integrated aTc-inducible eCFP expression cassette was used. Overnight bacterial cultures were diluted 1:50 into LB medium in 16-ml tubes supplemented with aTc (25 ng/ml) to induce eCFP expression. Cultures were incubated at 37°C with shaking at 280 rpm for 3 hours to allow adequate eCFP production. After induction, cells were harvested by centrifugation at 5000*g* for 5 min at room temperature, washed once with an equal volume of 3-hour spent LB medium (prepared by collecting the supernatant of *Salmonella* culture grown for 3 hours) to remove residual aTc, and resuspended in the same volume of 3-hour spent LB medium. The cultures were further incubated at 37°C in 96-well plates with shaking to allow continuous monitoring of cell growth and fluorescence. At the various incubation times, cells were collected by centrifugation and prepared for fluorescence microscopy, as described above.

### RNA/protein ratio determination

Total RNA and protein contents were quantified using a modified method from You *et al.* (*38*). Cells cultured in LB at 37°C for 3 or 22 hours were harvested and flash-frozen. For RNA extraction, pellets were washed with 0.7 M HClO_4_ and digested in 0.3 M KOH at 37°C with intermittent mixing. The lysate was neutralized with 0.1 ml of 3 M HClO_4_, and the precipitate was re-extracted twice with 0.5 M HClO_4_. Pooled supernatants were then analyzed using a Synergy HTX reader. For protein quantification, pellets were digested in 3 M NaOH at 98°C for 5 min. The Biuret reaction was initiated with 1.6% CuSO_4_ for 5 min at room temperature. Following centrifugation (13,000*g* for 1 min), the supernatant was measured at 555-nm absorbance.

### Polysome profiling

Cells cultured in LB medium at 37°C for 3 or 22 hours were treated with chloramphenicol (100 μg/ml) and incubated on ice for 3 min to arrest translation. Cells were harvested by centrifugation at 4000 rpm for 10 min, washed twice, and resuspended in lysis buffer [20 mM tris-HCl, pH 8, 140 mM KCl, 40 mM MgCl_2_, 0.5 mM dithiothreitol, 1% Triton X-100, chloramphenicol (100 μg/ml), and heparin (1 mg/ml)]. Cell lysis was achieved by grinding in a mortar and pestle under liquid nitrogen, followed by clarification at 8000 rpm. Total RNA (>150 μg) was loaded onto 10 to 40% (w/v) sucrose gradients prepared in buffer containing 20 mM tris-HCl, pH 8, 140 mM KCl, and 40 mM MgCl_2_. Gradients were formed by diffusion by incubating horizontally at room temperature for 4 hours and then vertically at 4°C overnight. Ultracentrifugation was performed at 35,000 rpm for 2.5 hours at 4°C using an SW40-Ti rotor (Beckman Coulter). Gradients were fractionated using a Teledyne ISCO fractionation system by upward displacement with perfluorotributylamine, and absorbance was continuously monitored at 254 nm.

### *Salmonella* infection assay

HeLa epithelial cells and J774A.1 macrophages were seeded into 12-well plates at a density of 5 × 10^5^ cells per well and cultured for 18 hours before use. Infections were performed using early-stationary-phase bacterial cultures (~5-hour growth) at a multiplicity of infection of 50. Infection was initiated by incubating the plates at 37°C with 5% CO_2_ for 25 min. For the attachment and invasion assay, the supernatant containing unbound bacteria was collected. The monolayers were washed three times with sterile PBS and lysed with 0.1% Triton X-100 to recover adherent bacteria. The lysate was first centrifuged at 700*g* for 5 min to remove mammalian cell debris. The resulting supernatant was then centrifuged at 5000*g* for 5 min to pellet the bacteria. Bacterial pellets were fixed with 4% paraformaldehyde for 10 min and imaged using a Keyence BZ-X800 fluorescence microscope. For the macrophage replication assay, extracellular bacteria were eliminated by incubating the monolayers in Dulbecco’s modified Eagle’s medium containing gentamicin (100 μg/ml) for 1 hour, and the infection was maintained in medium containing gentamicin (10 μg/ml) for another 17 hours. Last, intracellular bacteria were harvested and concentrated using the same lysis and differential centrifugation protocol described above for the attachment assay.

### Quantification and statistical analysis

All experiments were performed with at least three independent biological replicates. All images shown are representatives of the biological replicates. Standard errors for the ratio of protein abundances (as in Fig. 8) were obtained by propagating the standard errors for the protein abundances in the ratio, i.e., if R=A/B, then $\sigma_{R}^{2}/R^{2}=\sigma_{A}^{2}/A^{2}+\sigma_{B}^{2}/B^{2}$. Statistical significance was assessed using either a one-way analysis of variance (ANOVA) followed by Dunnett’s post hoc test or unpaired *t* tests with Welch’s correction. Significance thresholds were defined as follows: *P < 0.05, **P < 0.01, ***P < 0.001, and ****P < 0.0001.
