# Supplementary material for: Single-cell heterogeneity in ribosome levels and protein synthesis during nutrient starvation is driven by cAMP signaling
Source: Sci Adv. 2026 Jun 26;12(26):eaed2171. doi: 10.1126/sciadv.aed2171 (PMC13308603; doi:10.1126/sciadv.aed2171)
Supplement: Supplementary file 1 — Figs. S1 to S30 Table S1 Legends for tables S2 to S4 [file sciadv.aed2171_sm.pdf]

Science Advances
AAAS

Supplementary Materials for

Single-cell heterogeneity in ribosome levels and protein synthesis during nutrient starvation is driven by cAMP signaling

Zhihui Lyu *et al.*

Corresponding author: Jiqiang Ling, jling12@umd.edu

Sci. Adv. **12**, eaed2171 (2026)
DOI: 10.1126/sciadv.aed2171

The PDF file includes:

Figs. S1 to S30
Table S1
Legends for tables S2 to S4

Other Supplementary Material for this manuscript includes the following:

Tables S2 to S4

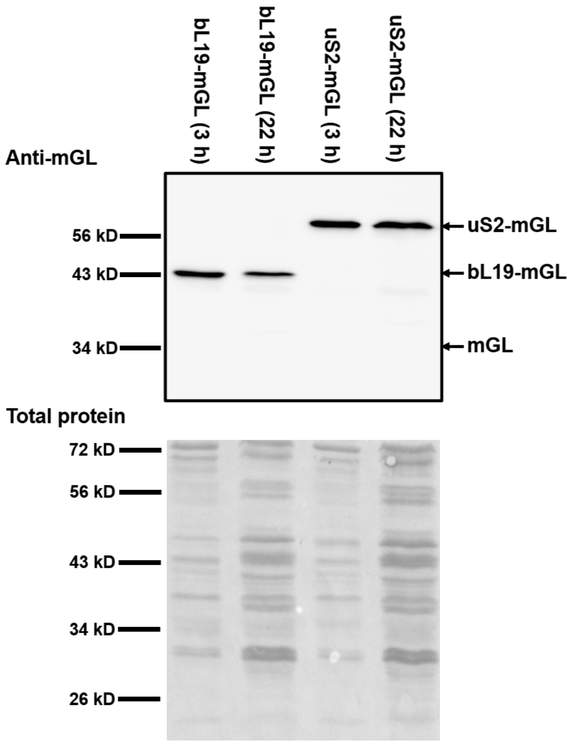

Fig. S1. **Western blot against mGL-labeled RPs, related to Fig. 1.** *Salmonella* cells were grown in LB at 37°C and harvested at 3 h and 22 h. The sizes of the fusion proteins were as expected, and no cleaved mGL was observed. The images are representative of three biological replicates.

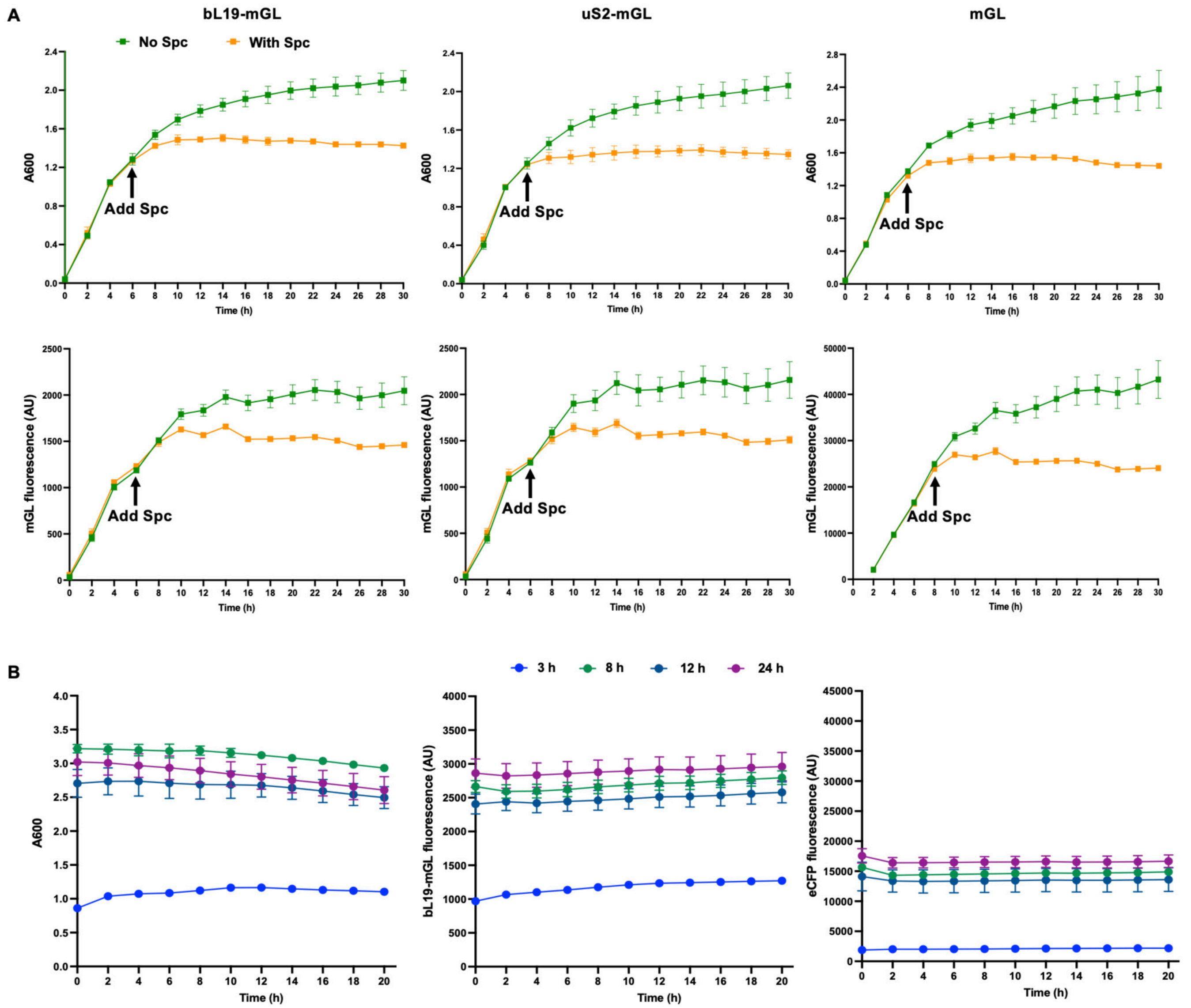

Fig. S2. **Stability of fluorescent proteins, related to Fig. 1.** *Salmonella* cells were grown in LB at 37°C. (A) Spc (300 μg/ml) was added at 6 h to inhibit protein synthesis and growth. bL19-mGL, uS2-mGL, and freestanding mGL were stable over time. (B) Spc (300 μg/ml) was added at various time points in the bL19-mGL strain carrying pZS-P*tet*-eCFP. Error bars represent one standard deviation from the means (n = 4).

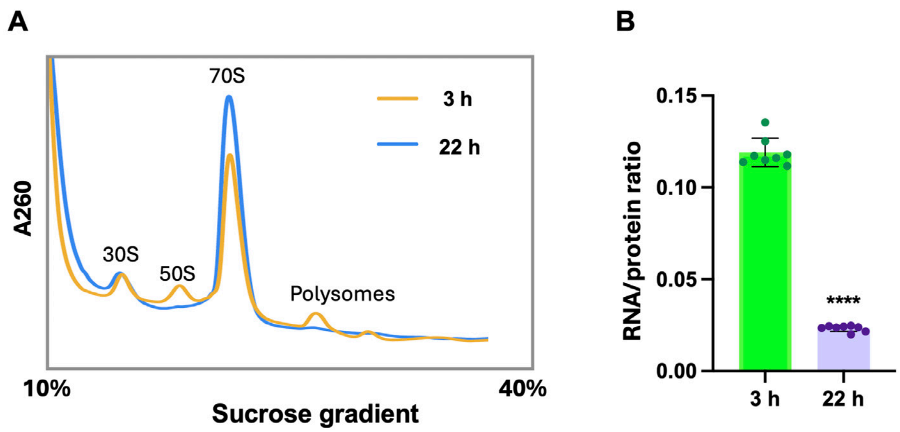

Fig. S3. **Polysome profiles and RNA/protein ratio, related to Fig. 1.** The bL19-mGL *Salmonella* cells were grown in LB at 37°C to 3 or 22 h and analyzed for (A) the polysome profile and (B) the RNA/protein ratio, which is a proxy for the rRNA content as rRNAs account for the majority of cellular RNAs (*43*). The polysome profiles are representatives of three biological replicates. Error bars represent one standard deviation from the means. The P value is calculated using the unpaired t-test with Welch's correction. **** P < 0.0001.

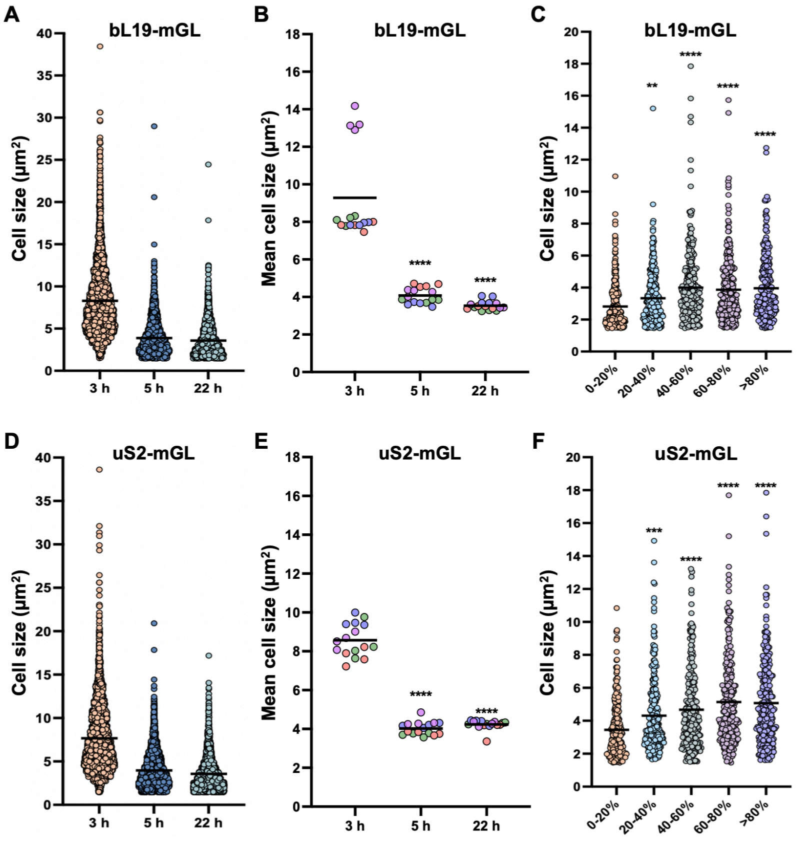

Fig. S4. **Correlation between cell sizes and RP levels, related to Fig. 1.** (A, D) Dot plots of cell sizes at various growth stages. (B, E) Mean cell sizes. Biological replicates are indicated in different colors, and technical replicates are shown in the same colors. (C, F) Bin analysis of cell sizes based on the RP rankings from low to high at 22 h. The bin analyses are representatives of at least three biological replicates. The P values are calculated using one-way ANOVA with Dunnett's test comparing other subpopulations with the lowest RP subpopulation (0-20%). **** P < 0.0001.

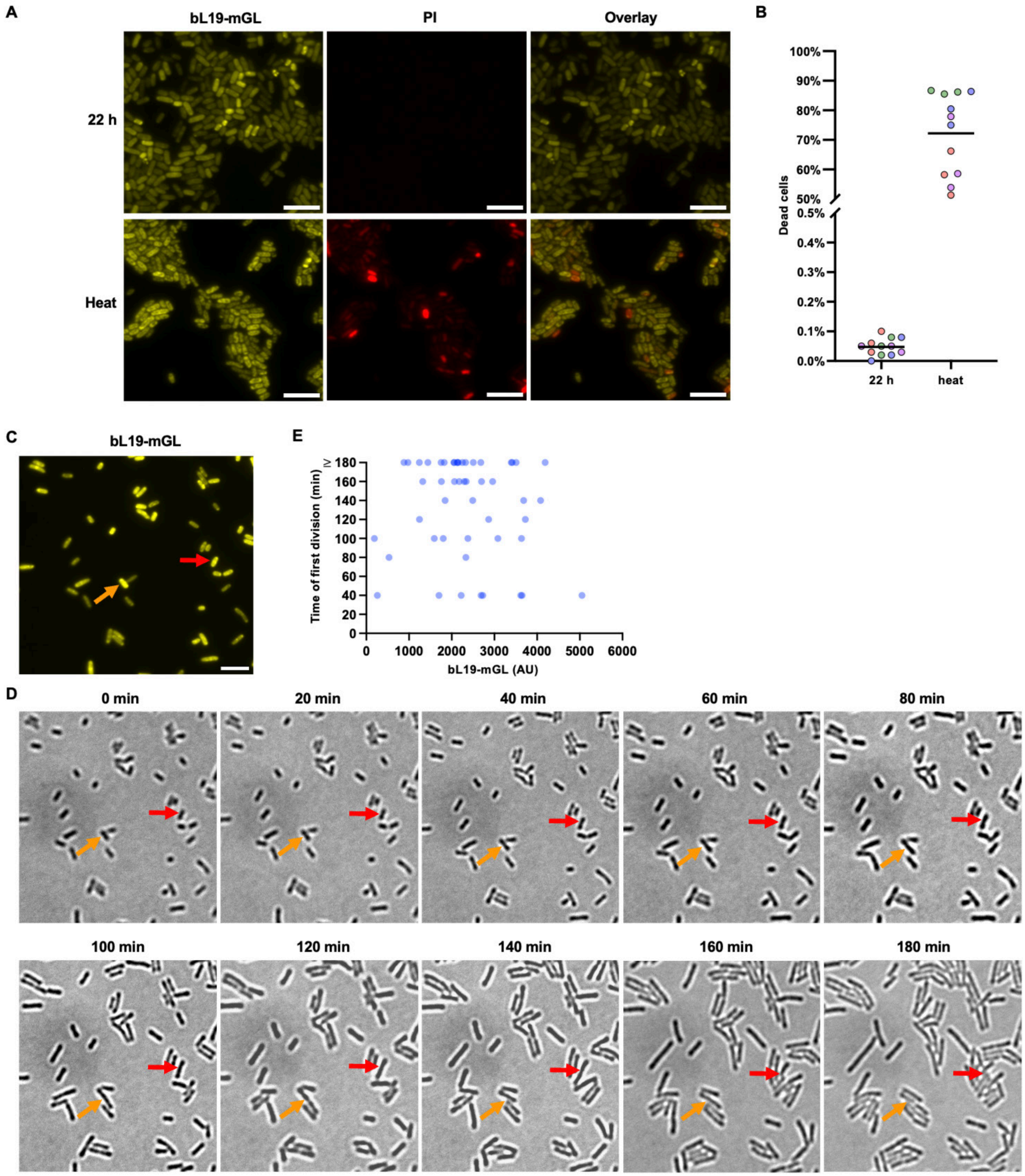

Fig. S5. Viability and regrowth of stationary-phase cells, related to Fig. 1. (A) Propidium iodide (PI) staining of *Salmonella* cells grown in LB at 37°C for 22 h with and without subsequent heat treatment (60°C for 15 min). PI stains dead, membrane-permeable cells. (B) Quantitation of PI-stained dead cells. (C-E) Time-lapse microscopy of 22 h cells regrowing in fresh LB. (C) Fluorescence image at time 0. (D) Differential interference contrast (DIC) images over time. (E) Correlation between time to the first cell division in fresh LB and bL19-mGL fluorescence in single cells. The images are representative of at least two biological replicates. Scale bars: 5 µm.

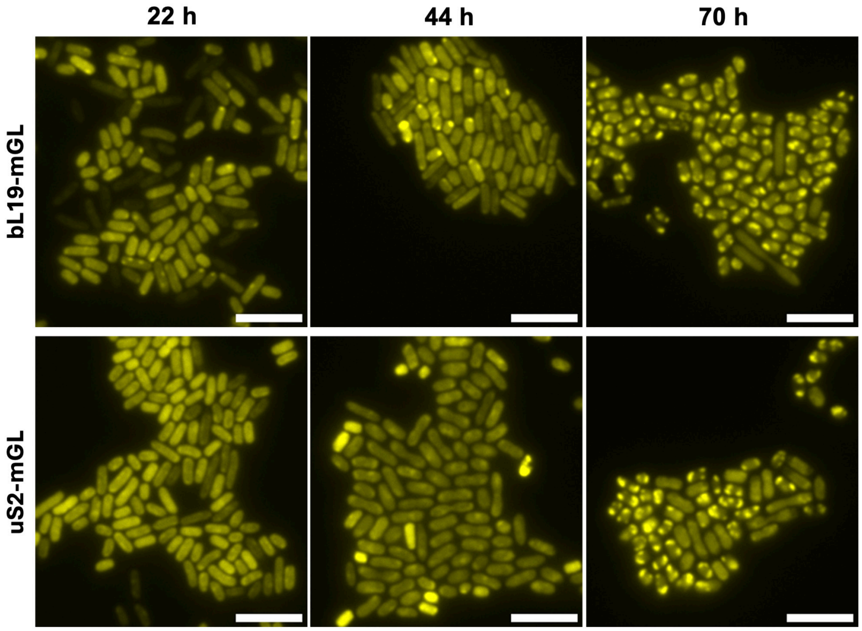

Fig. S6. **Fluorescence microscopy images of stationary-phase cells, related to Fig. 1.** *Salmonella* cells were grown in LB at 37°C for 22-70 h. bL19 and uS2 formed foci in the late stationary phase (70 h). The images are representative of three biological replicates. Scale bars: 5 µm.

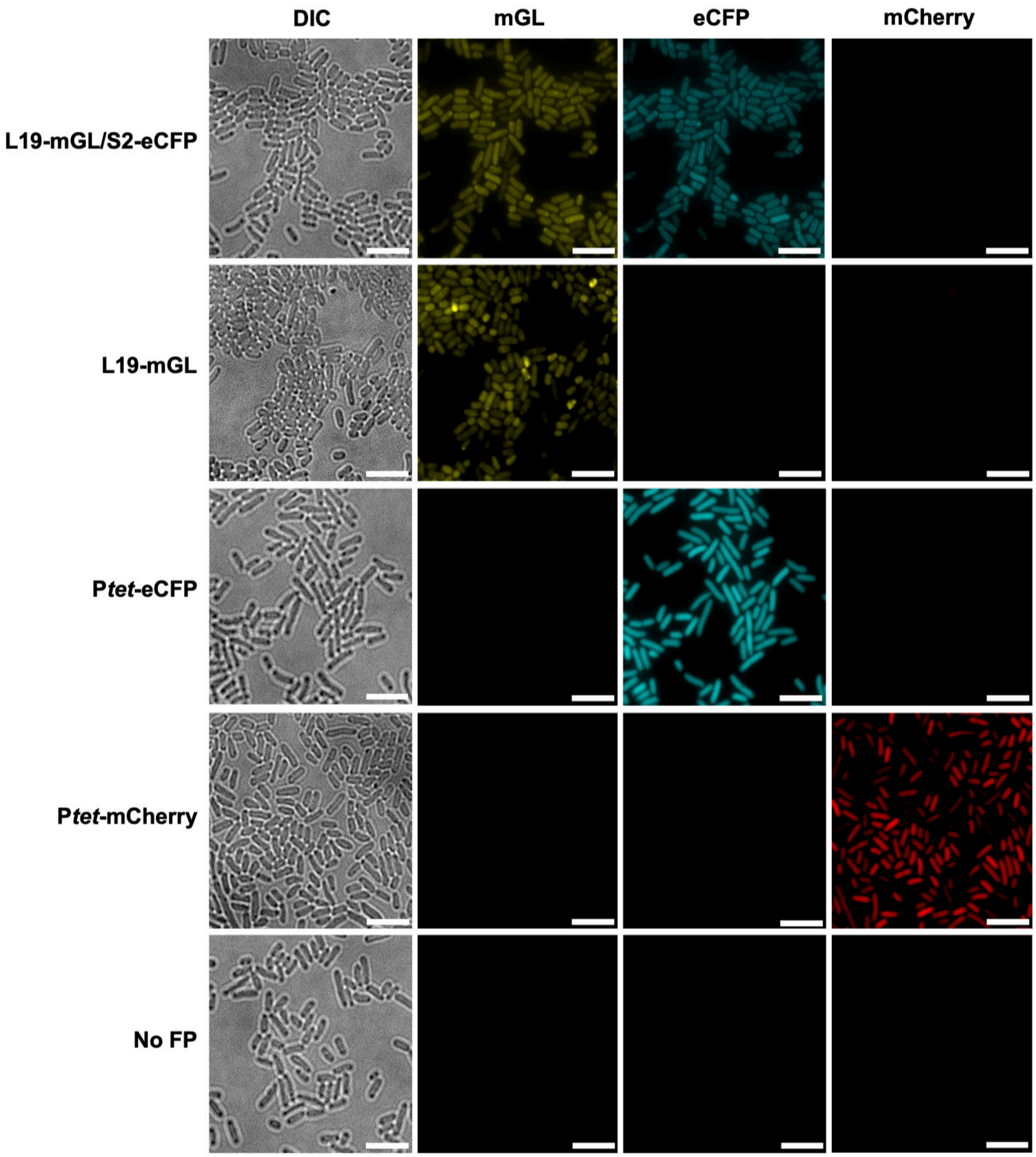

Fig. S7. Autofluorescence and crosstalk among mGL, eCFP, and mCherry, related to Fig. 1. *Salmonella* cells were grown in LB at 37°C for 22 h. No background fluorescence or crosstalk was observed under the experimental conditions. The images are representative of three biological replicates. Scale bars: 5 µm.

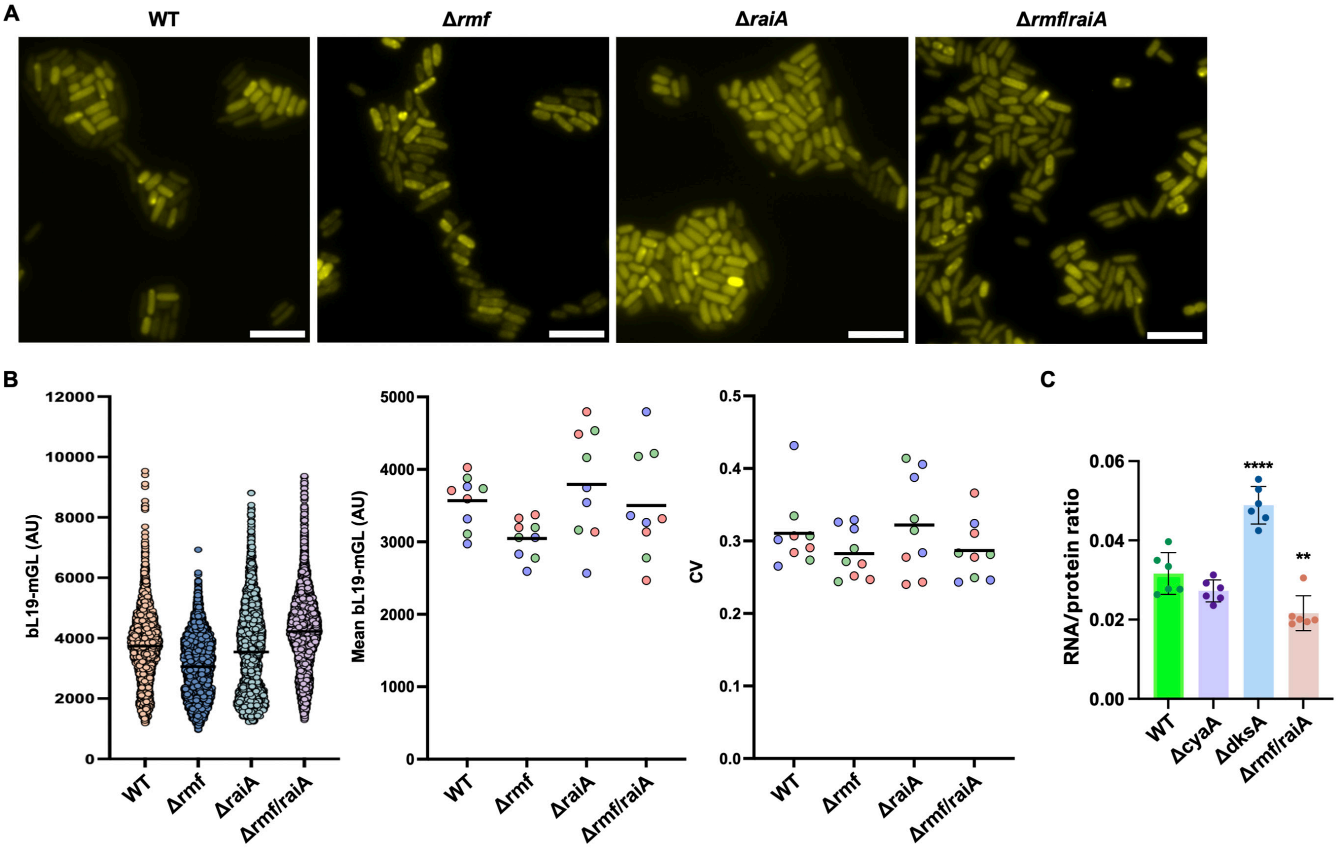

Fig. S8. Effects of ribosome hibernation factors on ribosome levels, related to Fig. 2. *Salmonella* cells were grown in LB at 37°C for 22 h. (A) Representative microscopy images of bL19-mGL fluorescence. (B) Quantitative analyses of fluorescence signals. Deleting *rmf* and *raiA* does not significantly affect the mean and heterogeneity of bL19 levels. For mean bL19-mGL and CV, biological replicates are indicated in different colors, and technical replicates are shown in the same colors. (C) The RNA/protein ratios. Error bars represent one standard deviation from the means. The P values are calculated using one-way ANOVA with Dunnett's test comparing mutants with the WT. ** $P < 0.01$; **** $P < 0.0001$. Scale bars: 5 μm.

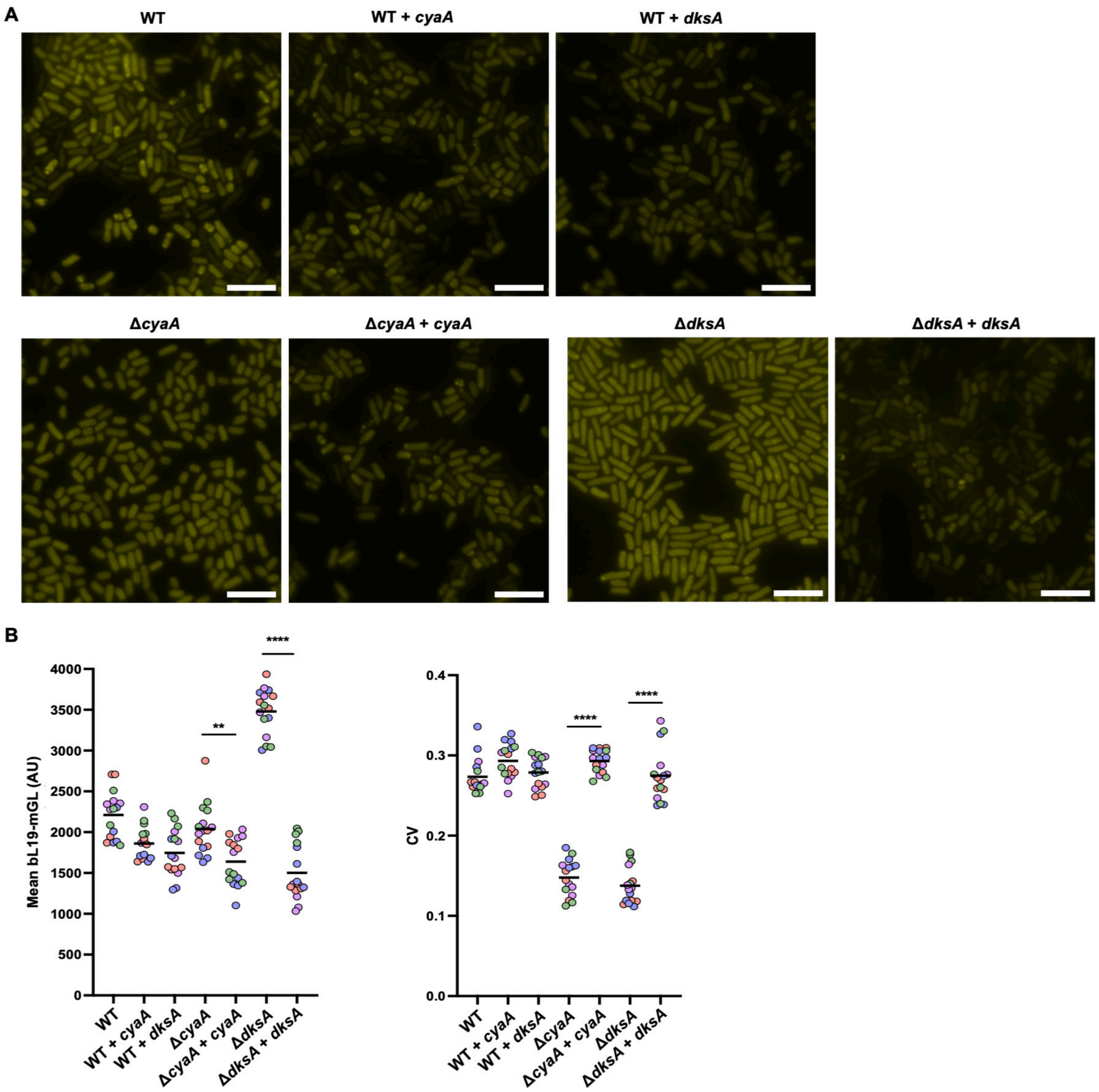

Fig. S9. **RP variations of *cyaA* and *dksA* complementation strains, related to Fig. 2.** (A) Representative fluorescence microscopy images of *Salmonella* cells grown in LB at 37°C at 22 h. (B) Means and heterogeneity of bL19 fluorescence in single cells. Biological replicates are indicated in different colors, and technical replicates are shown in the same colors. AU, arbitrary units. CV, coefficient of variance. The P values are calculated using two-way ANOVA with Tukey's post-hoc test. ** $P < 0.01$; **** $P < 0.0001$. Scale bars: 5 μm.

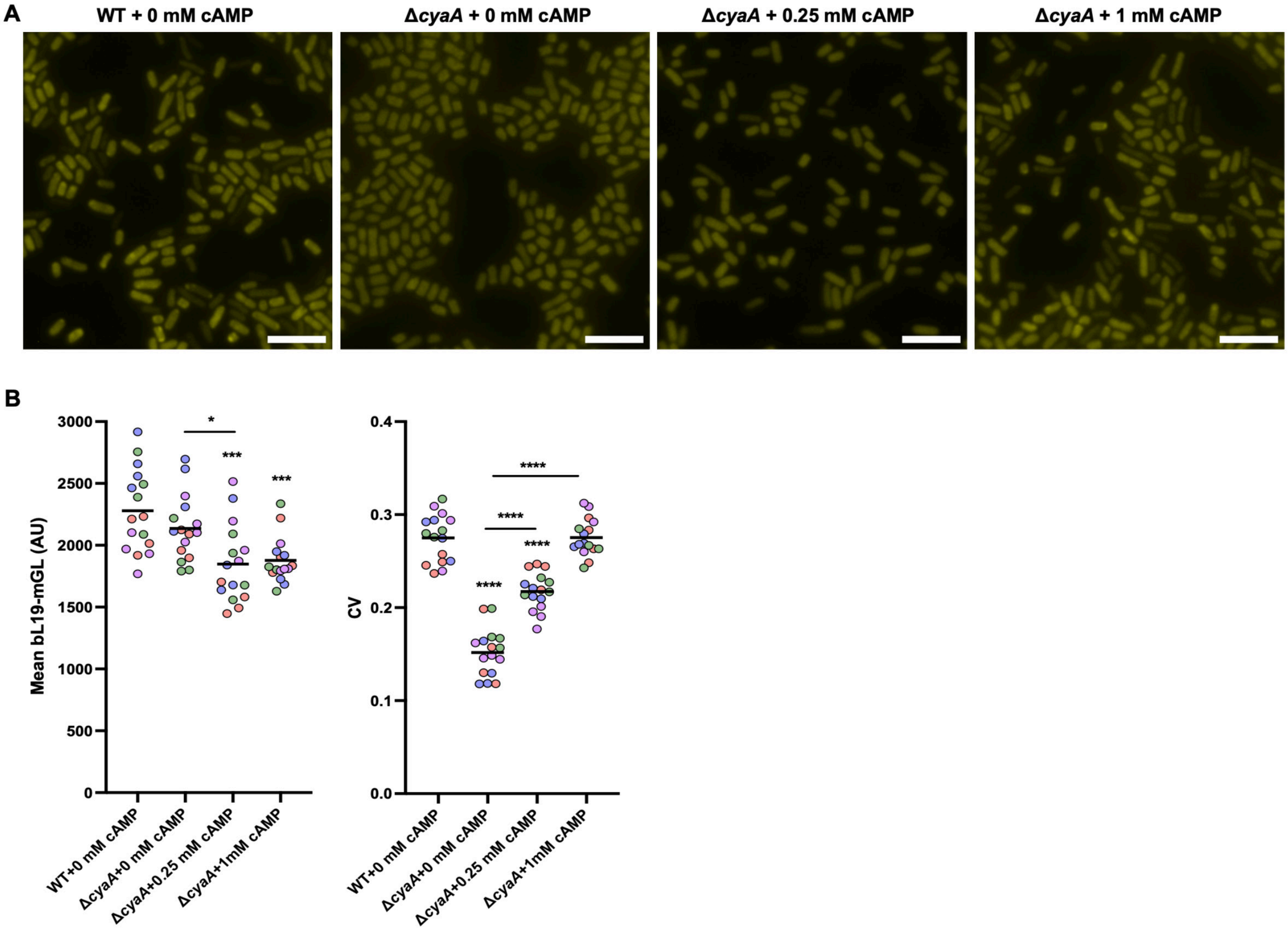

Fig. S10. Effects of cAMP supplementation on bL19-mGL levels, related to Fig. 2. *Salmonella* cells were grown in LB at 37°C for 22 h with or without cAMP added at 0 h. (A) Representative microscopy images of bL19-fluorescence. (B) Quantitative analyses of fluorescence signals. Biological replicates are indicated in different colors, and technical replicates are shown in the same colors. The P values are calculated using one-way ANOVA with Dunnett's test. * $P < 0.05$; *** $P < 0.001$; **** $P < 0.0001$. Scale bars: 5 μm.

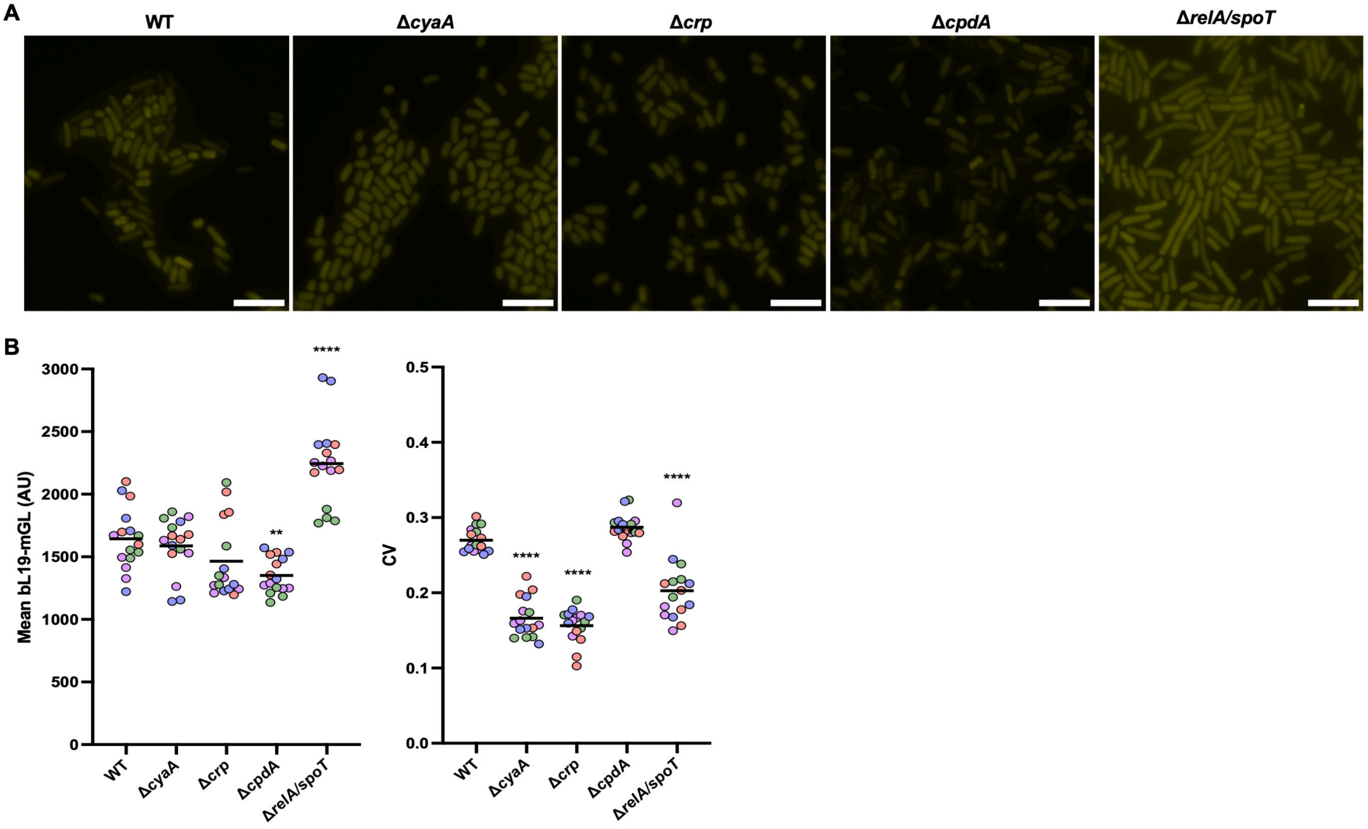

Fig. S11. **RP variations of *Salmonella* strains, related to Fig. 2.** (A) Representative fluorescence microscopy images of *Salmonella* cells grown in LB at 37°C at 22 h. (B) Means and heterogeneity of bL19 fluorescence in single cells. Biological replicates are indicated in different colors, and technical replicates are shown in the same colors. AU, arbitrary units. CV, coefficient of variance. The P values are calculated using one-way ANOVA with Dunnett's test compared with the WT. **** $P < 0.0001$. Scale bars: 5 μm.

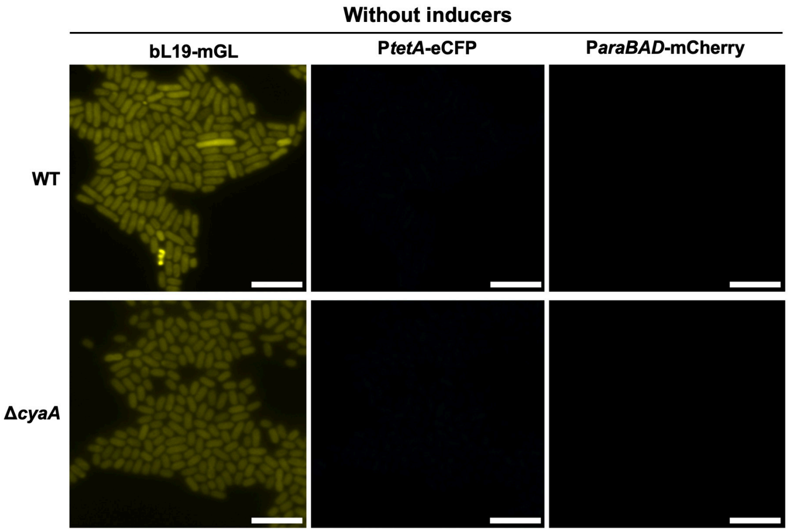

Fig. S12. Background fluorescence of the inducible dual-reporter system for cAMP activity, related to Fig. 3. pZS P*tetA*-eCFP P*araBAD*-mCherry was expressed in the bL19-mGL strain in LB without inducers (aTc and L-arabinose). No eCFP or mCherry fluorescence was observed with the same exposure time as in Fig. 3. The images are representatives of three biological replicates. Scale bars: 5 μm.

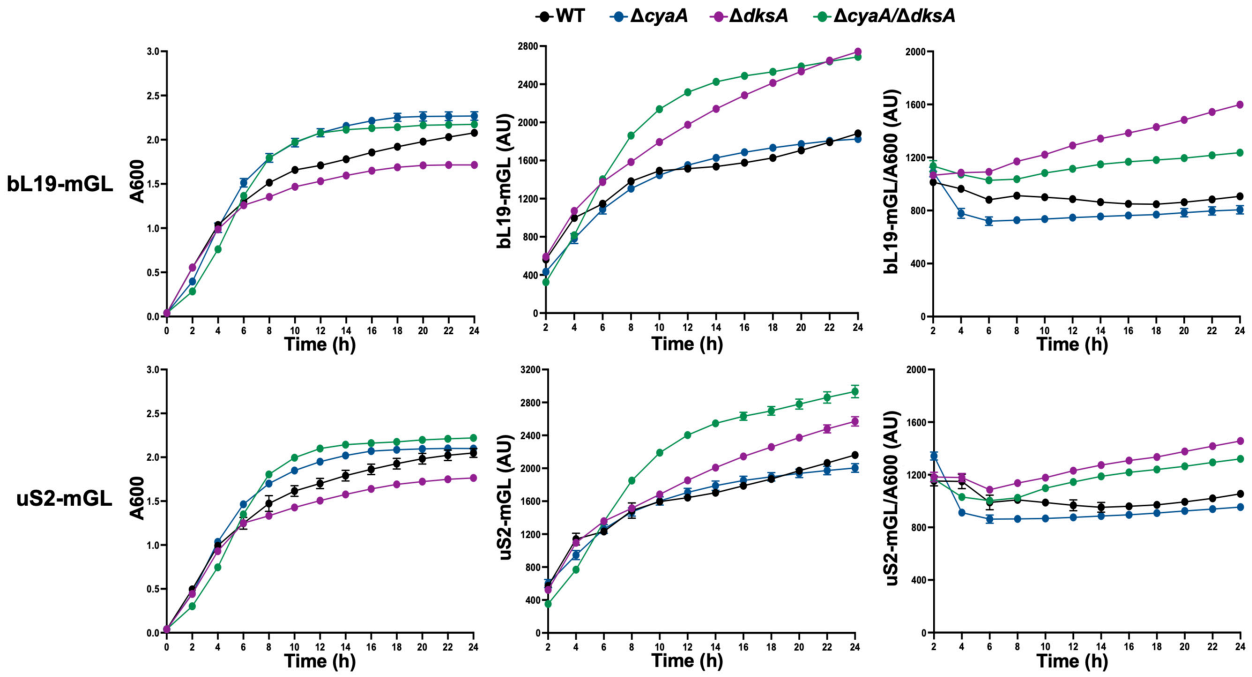

Fig. S13. Growth and fluorescence of *Salmonella* variants, related to Fig. 2. Error bars represent one standard deviation from the means (n = 4).

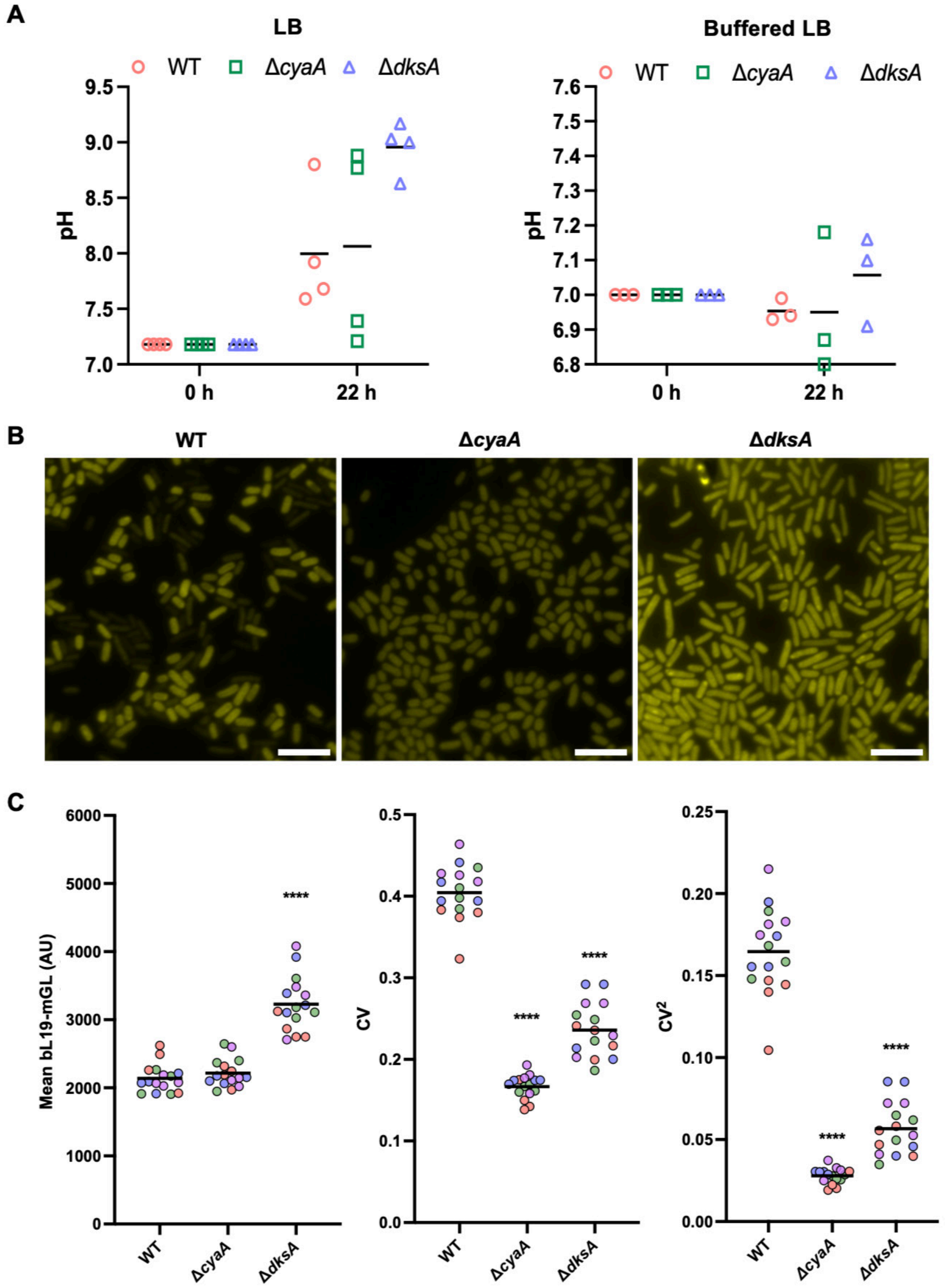

Fig. S14. **Heterogeneity of RP levels in buffered LB, related to Fig. 2.** (A) Medium pH of *Salmonella* cultures grown in regular or buffered LB at 37°C. (B) Representative fluorescence microscopy images of *Salmonella* cells grown in buffered LB at 37°C at 22 h. (C) Means and heterogeneity of bL19 fluorescence in single cells. In (C), biological replicates are indicated in different colors, and technical replicates are shown in the same colors. AU, arbitrary units. CV, coefficient of variance. The P values are calculated using one-way ANOVA with Dunnett's test compared with the WT. **** $P < 0.0001$. Scale bars: 5 μm.

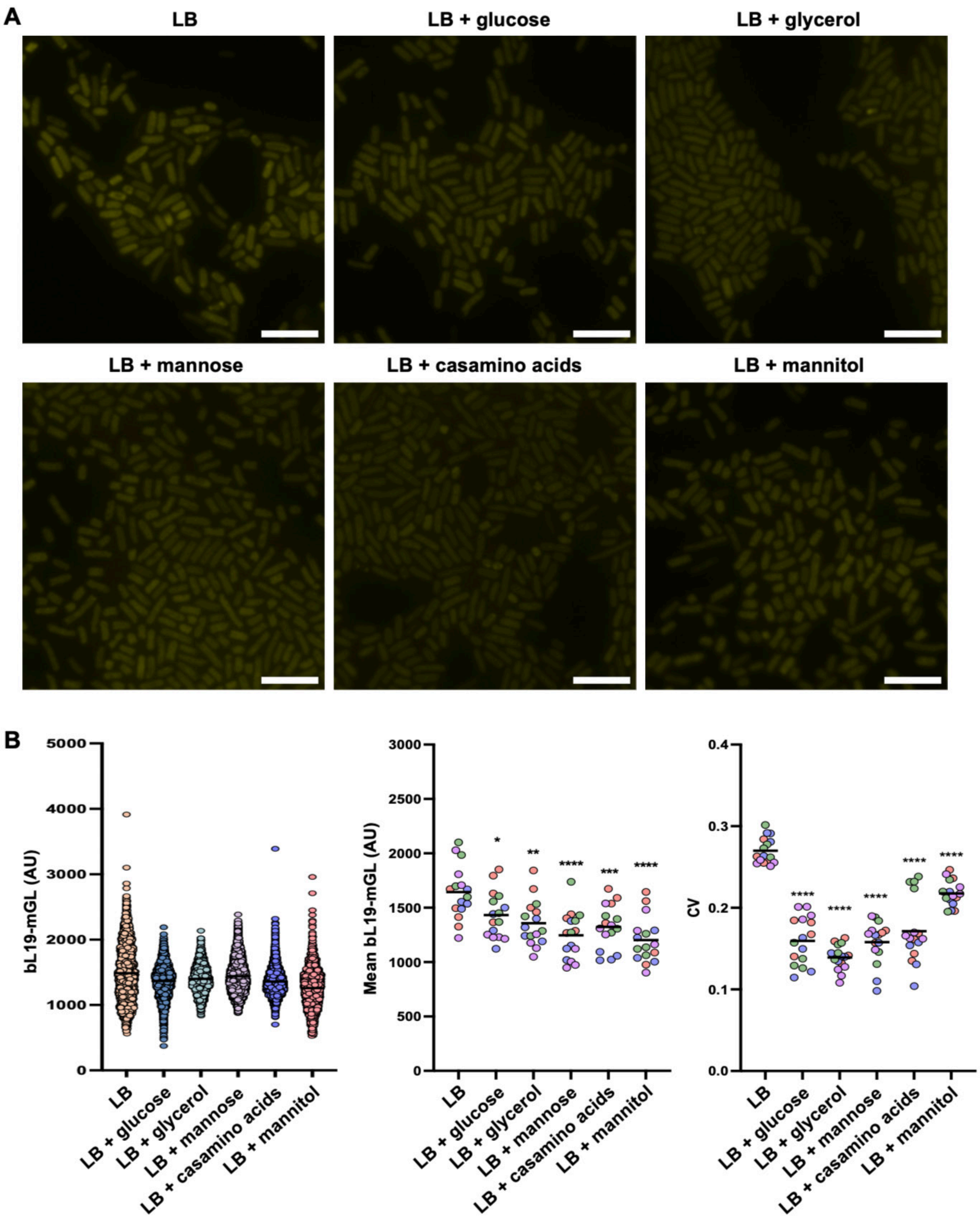

Fig. S15. **Effects of additional carbon sources on RP variations, related to Fig. 2.** WT bL19-mGL *Salmonella* cells were grown in LB at 37°C for 5 h, and 0.4% of additional carbon sources were added. The cells were further grown for 17 h before imaging. (A) Representative fluorescence microscopy images. (B) Quantitation of bL19 fluorescence in single cells. Biological replicates are indicated in different colors, and technical replicates are shown in the same colors. AU, arbitrary units. CV, coefficient of variance. The mean and CV of the WT control are the same as those in Fig. S11, as the data in Figs. S11 and S15 are from the same experiments. The P values are calculated using one-way ANOVA with Dunnett's test compared with samples in LB with no additional carbon source. * $P < 0.05$; ** $P < 0.01$; *** $P < 0.001$; **** $P < 0.0001$. Scale bars: 5 µm.

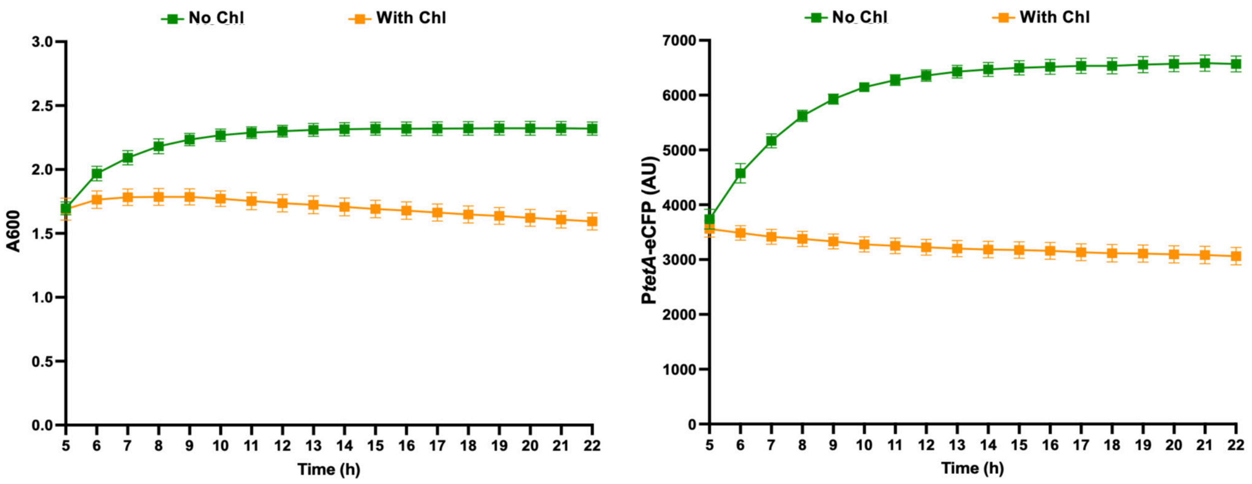

Fig. S16. Stability of eCFP in *Salmonella*, related to Fig. 5. Chl was used to inhibit protein synthesis. Error bars represent one standard deviation from the means (n = 4).

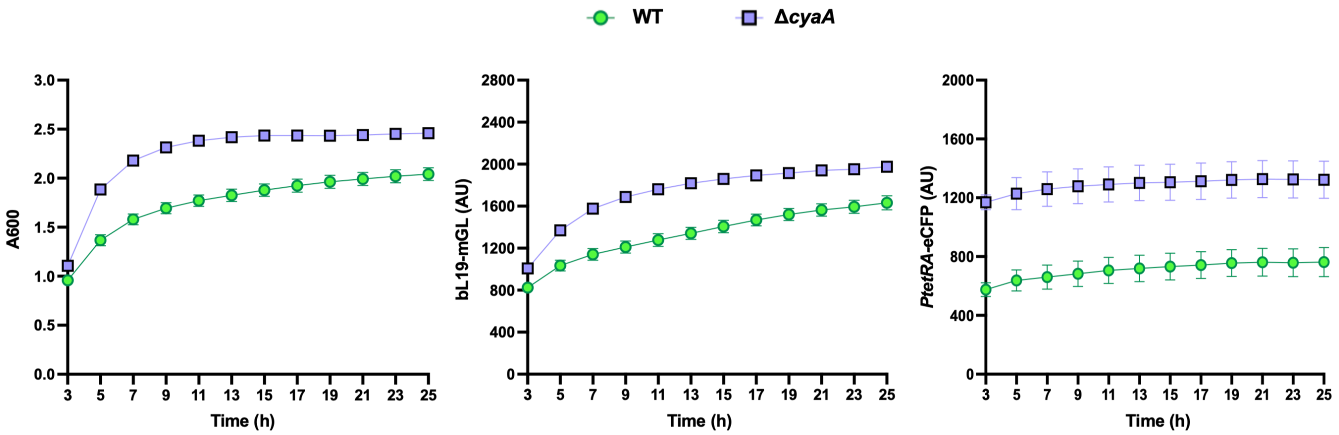

Fig. S17. Platereader data of the fluorescence dilution assay, related to Fig. 5. Chromosomal P*tetA*-eCFP was induced with 25 ng/ml aTc for 3 h. The inducer aTc was then removed by medium exchange, and the cells were further cultured in 96-well plates. (A) the Δ*cyaA* mutant gains a higher A600 than the WT. (B) Both the WT and Δ*cyaA* strains continued to increase bL19-mGL levels in the population after aTc was removed. (C) Little eCFP was made after 5 h (2 h after aTc was removed). The plots and error bars represent the mean and standard deviations of four biological replicates.

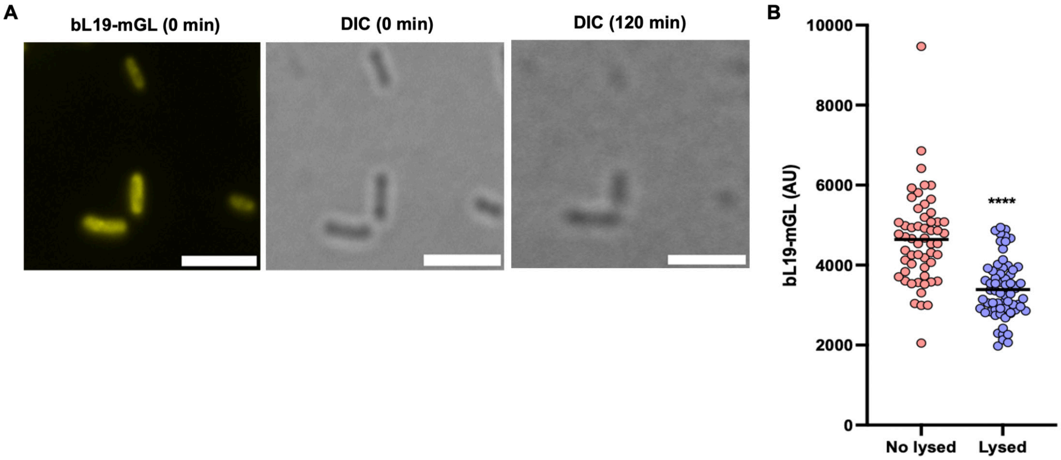

Fig. S18. Susceptibility of individual *Salmonella* cells to ampicillin, related to Fig 5. WT bL19-mGL cells were grown in LB at 37°C for 2 h. The cells were placed on LB agarose pads with 250 μg/ml ampicillin and imaged at various time points. (A) Representative fluorescence and DIC images of three biological replicates. (B) bL19-mGL fluorescence in single cells that were either lysed or not lysed in the presence of ampicillin by 120 min. The P value is calculated using the unpaired t-test with Welch's correction. **** P < 0.0001.

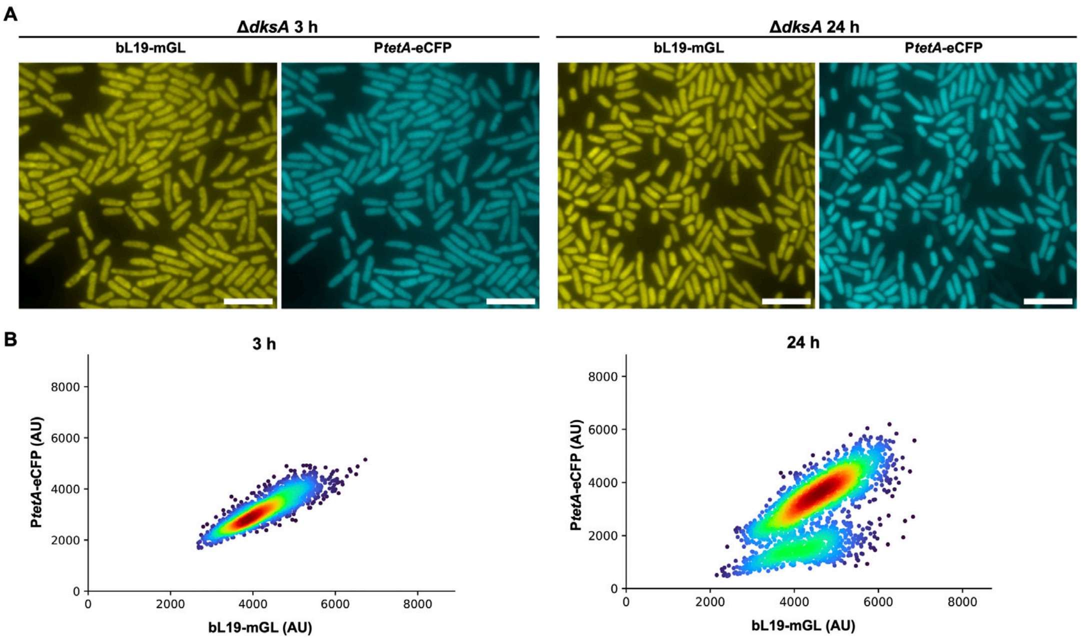

Fig. S19. Fluorescence dilution in Δ*dksA*, related to Fig. 5. (A) Chromosomal P*tetA*-eCFP was induced with 25 ng/ml aTc for 3 h. The inducer aTc was then removed by medium exchange, and the cells were further cultured to 24 h. (B) Scatter plots of P*tetA*-eCFP vs bL19-mGL. The images are representatives of at least three biological replicates.

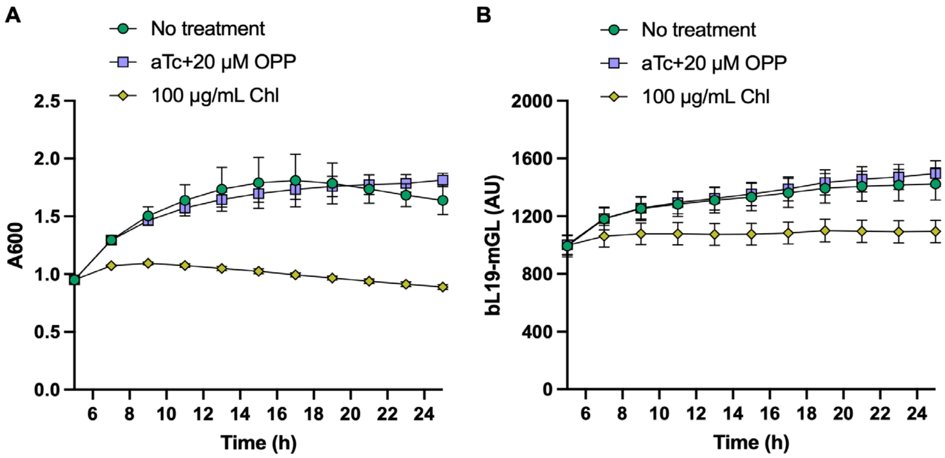

Fig. S20. Effects of aTc and OPP on growth and bL19 synthesis, related to Fig 6. *Salmonella* cells were grown in LB at 37°C to 5 h. 100 μg/ml chloramphenicol (Chl) or 250 μg/ml aTc + 20 μM OPP were added. (A) A600 and (B) bL19-mGL fluorescence were measured in a platereader. Adding aTC and OPP did not affect growth or bL19 synthesis. In contrast, Chl stopped growth and bL19 synthesis. Error bars represent one standard deviation from the means (n = 4).

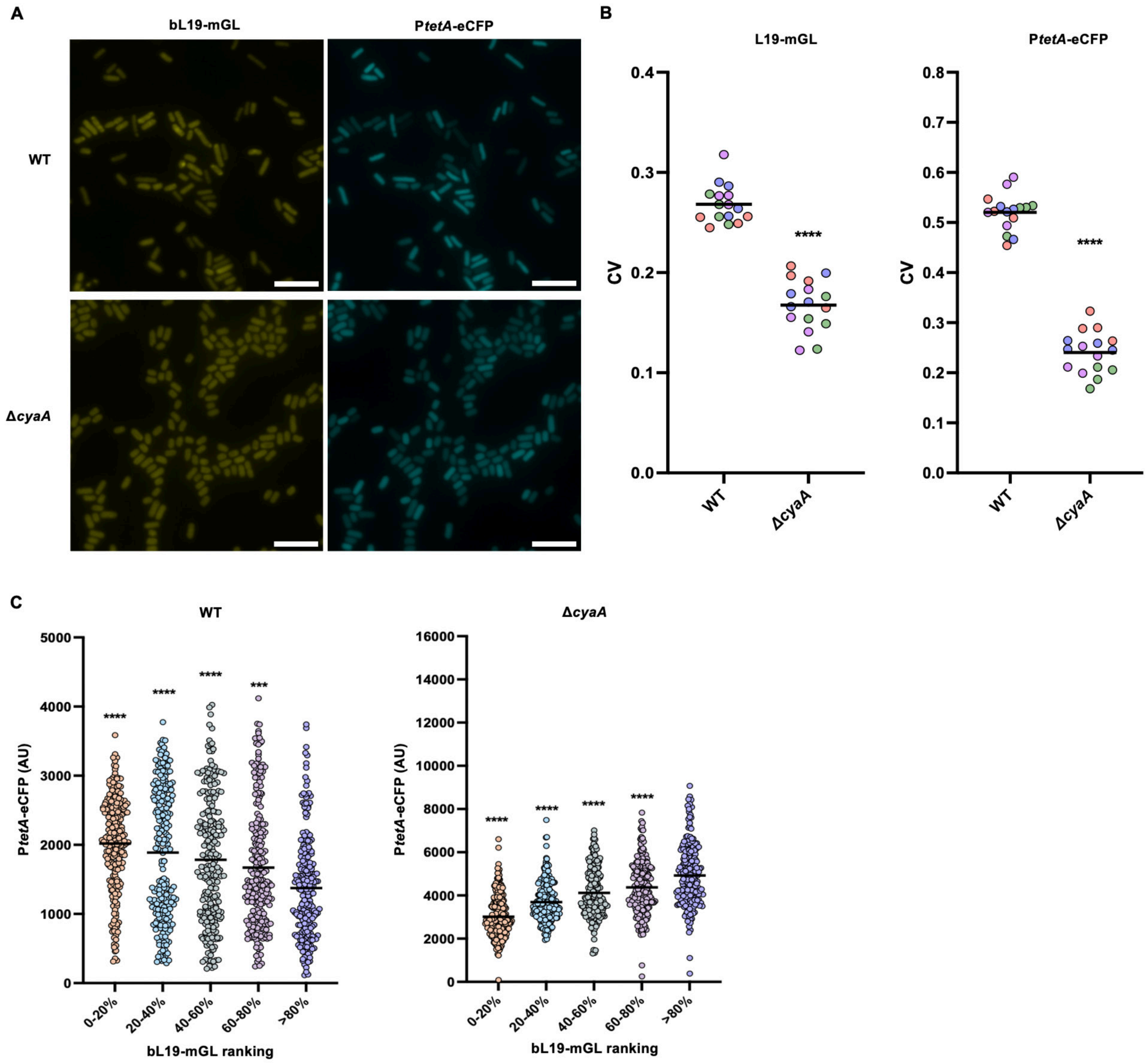

Fig. S21. Expression of chromosomal P*tetA*-eCFP, related to Fig 6. *Salmonella* cells carrying P*tetA*-eCFP on the chromosome were grown to 5 h and induced with 250 ng/ml aTc. (A) Representative fluorescence images. (B) CV of fluorescence signals. Biological replicates are indicated in different colors, and technical replicates are shown in the same colors. (C) Bin analyses of fluorescence signals based on the ranking of bL19-mGL levels from low to high. Like Fig. 6, the expression levels of P*tetA*-eCFP were negatively correlated with bL19-h levels in individual WT cells. The images and bin analyses are representatives of at least three biological replicates. The P values are calculated using one-way ANOVA with Dunnett's test compared with the >80% subpopulation. *** $P < 0.001$; **** $P < 0.0001$. Scale bars: 5 μm.

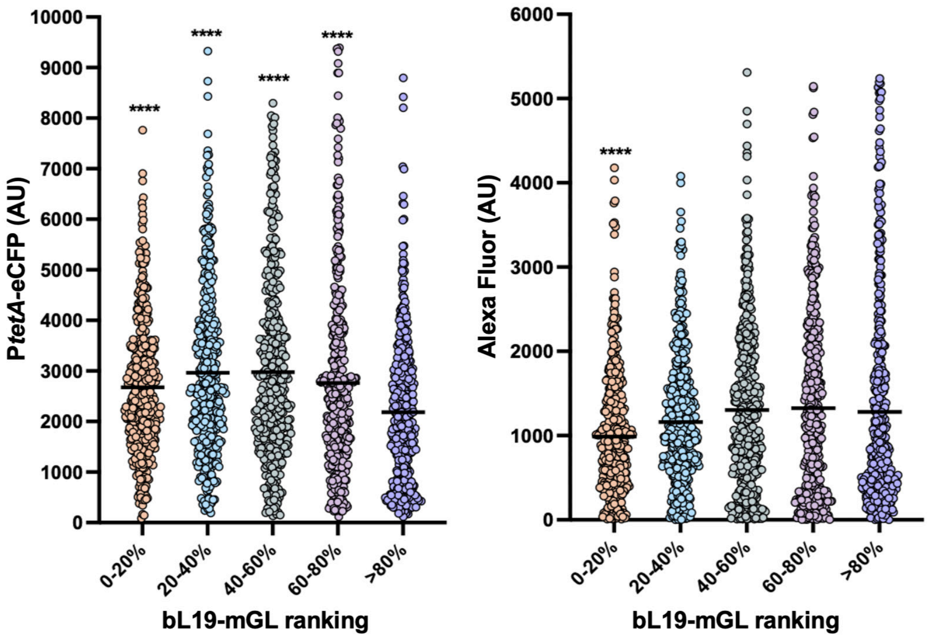

Fig. S22. Gene expression in Δ*rmf/raiA Salmonella*, related to Fig. 6. Cells were grown to 19 h and induced with 250 ng/ml aTc and 20 μM OPP for 3 h. Bin analyses of fluorescence signals were based on the ranking of bL19-mGL levels from low to high. The bin analyses are representatives of at least three biological replicates. The P values are calculated using one-way ANOVA with Dunnett's test compared with the >80% subpopulation. **** P < 0.0001.

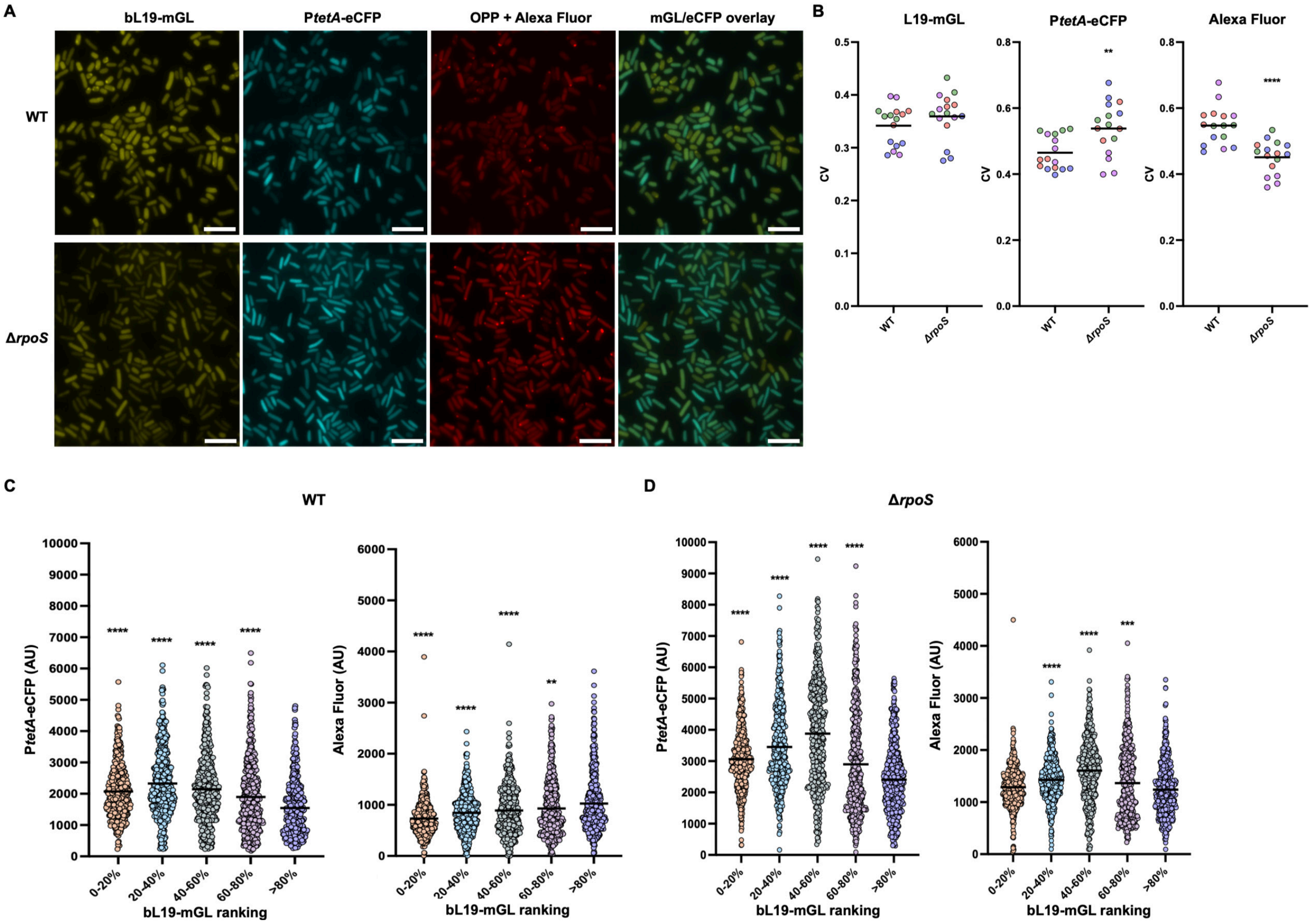

Fig. S23. Gene expression in the stationary-phase Δ*rpoS* cells, related to Fig 6. (A) Cells carrying pZS-Ptet-eCFP were grown to 19 h and induced with 250 ng/ml aTc and 20 μM OPP for 3 h. (B) CV of fluorescence signals. Biological replicates are indicated in different colors, and technical replicates are shown in the same colors. (C, D) Bin analyses of fluorescence signals based on the ranking of bL19-mGL levels from low to high. The images and bin analyses are representatives of at least three biological replicates. The P values are calculated using one-way ANOVA with Dunnett's test compared with the >80% subpopulation. ** $P < 0.01$; *** $P < 0.001$; **** $P < 0.0001$. Scale bars: 5 μm.

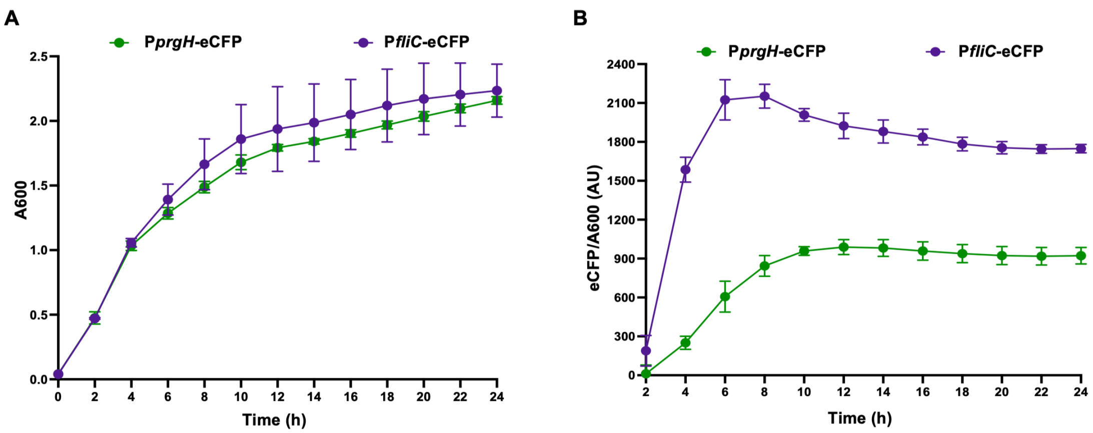

Fig. S24. Expression of virulence reporters over time, related to Fig. 7. (A) Growth curves and (B) A600-normalized fluorescence of WT *Salmonella* carrying pZS-P*prgH*-eCFP or pZS-P*fliC*-eCFP in LB at 37°C. Error bars represent one standard deviation from the means (n = 4).

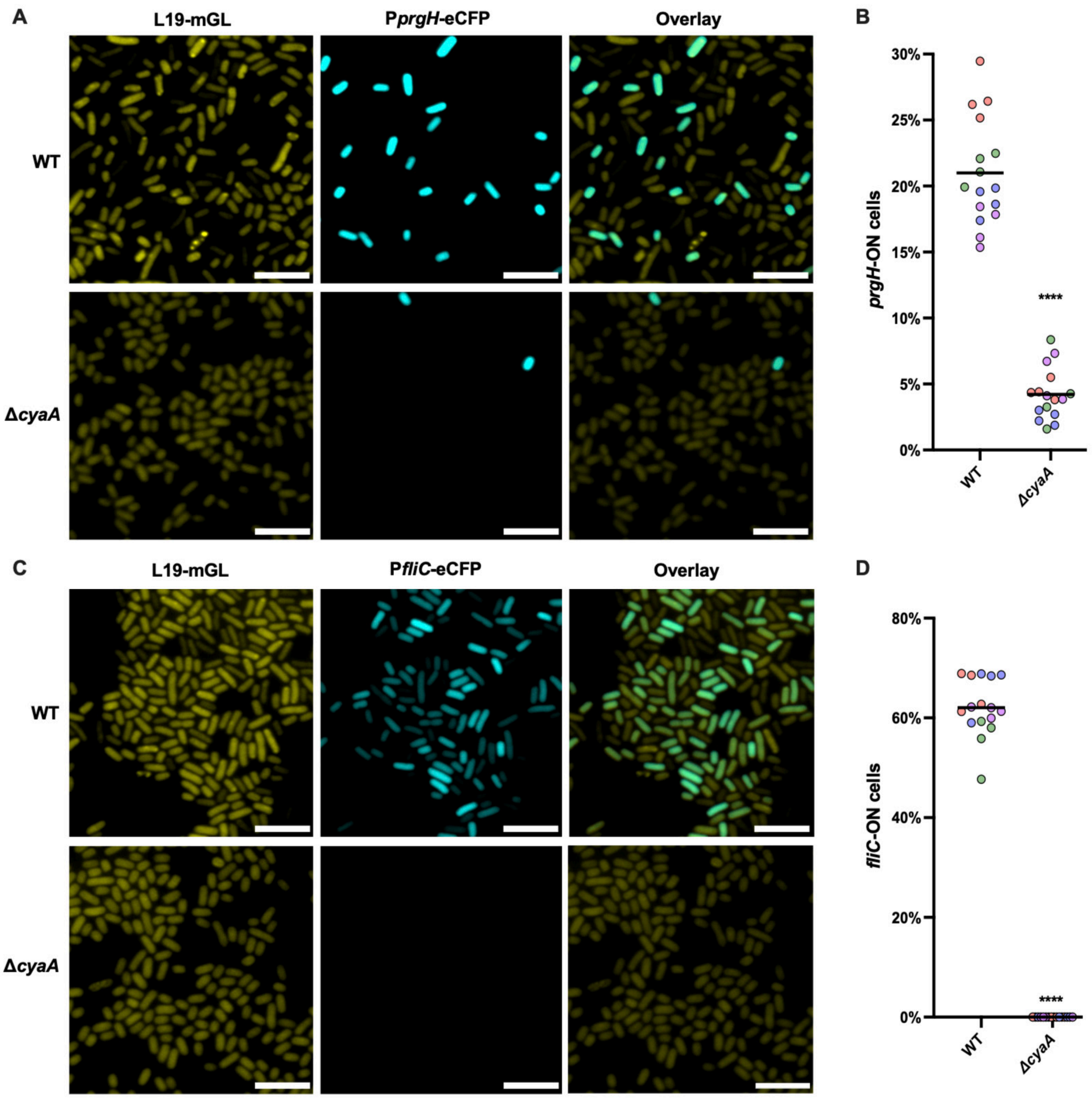

Fig. S25. Expression of SPI-1 and flagellar genes, related to Fig. 7. (A, C) Representative fluorescence microscopy images. (B, D) Percentage of SPI-1 (*prgH*-ON) and flagella (*fliC*-ON) positive cells. Biological replicates are indicated in different colors, and technical replicates are shown in the same colors. The P values are calculated using one-way ANOVA with Dunnett's test. **** $P < 0.0001$. Scale bars: 5 μm.

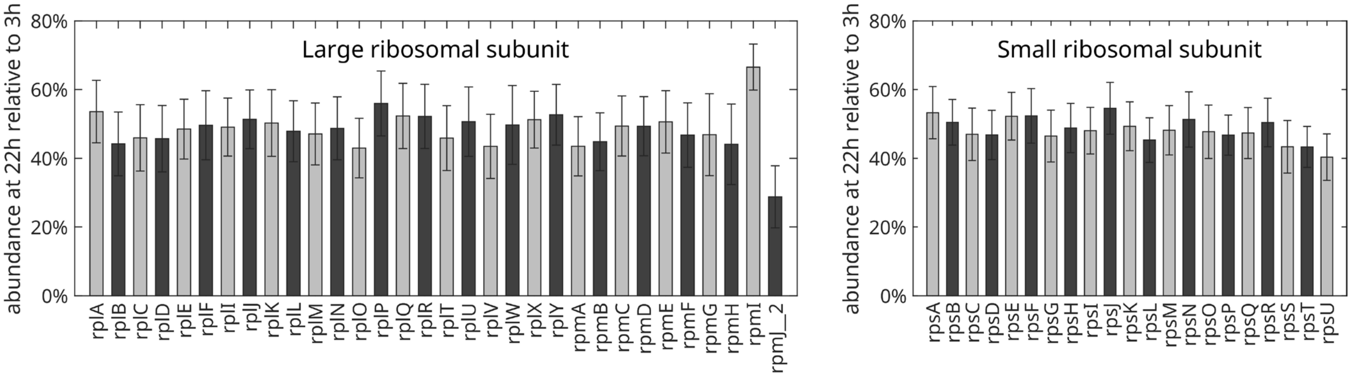

Fig. S26. Abundances of ribosomal proteins for WT strain at 22h, relative to the 3h timepoint, related to Fig. 8. The stationary-phase-induced RpsV protein is not shown; likewise, the RpmE2 protein was not detected at 22 h. Bars and whiskers are means and standard deviations (n = 3). Protein abundance data are taken from Table S3.

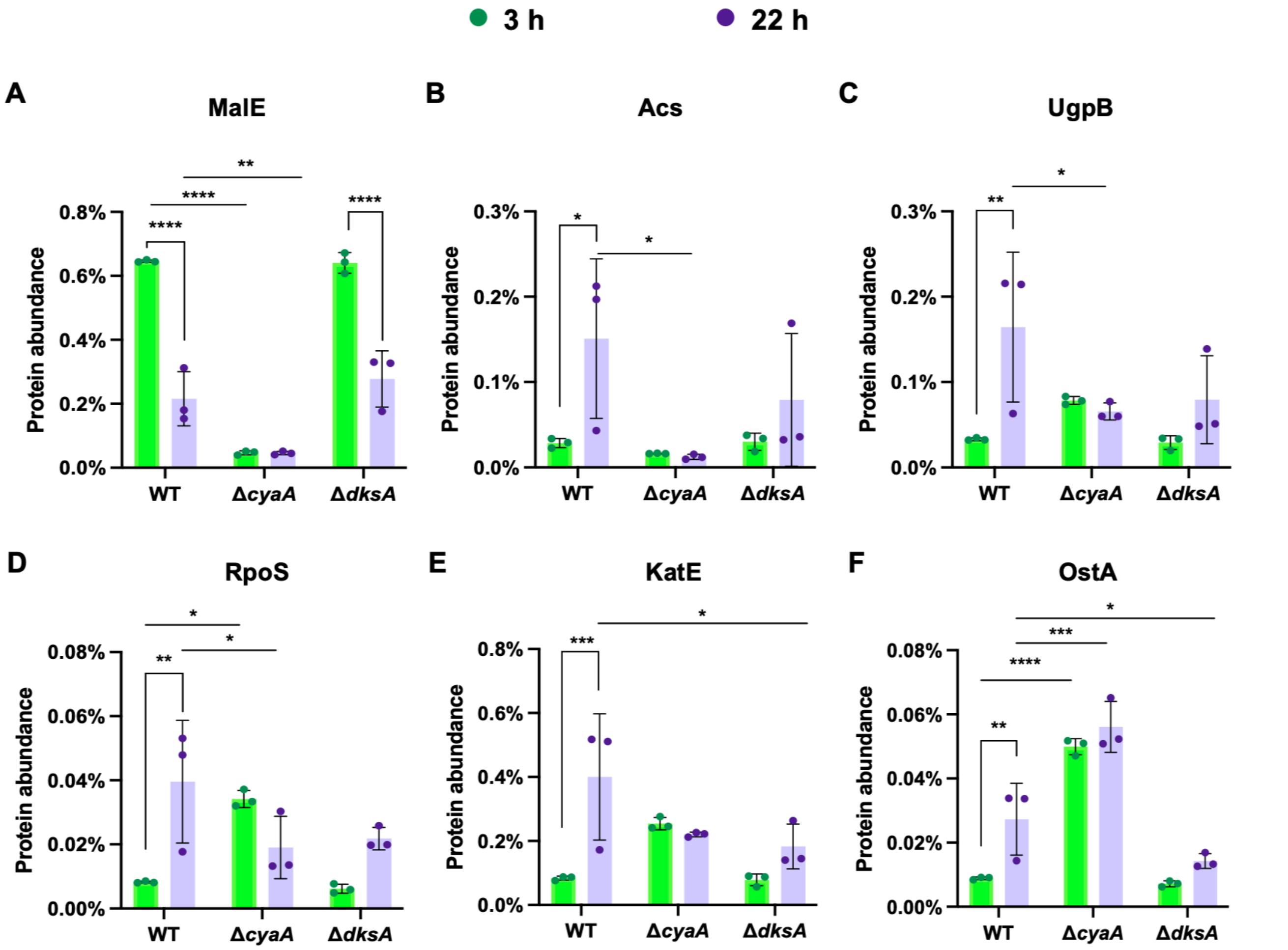

Fig. S27. Protein abundances of individual proteins across conditions, related to Fig. 8. The protein abundance data are taken from Table S3. Error bars represent one standard deviation from the means. The P values are calculated using two-way ANOVA with Tukey's post-hoc test. * $P < 0.05$; ** $P < 0.01$; *** $P < 0.001$; **** $P < 0.0001$.

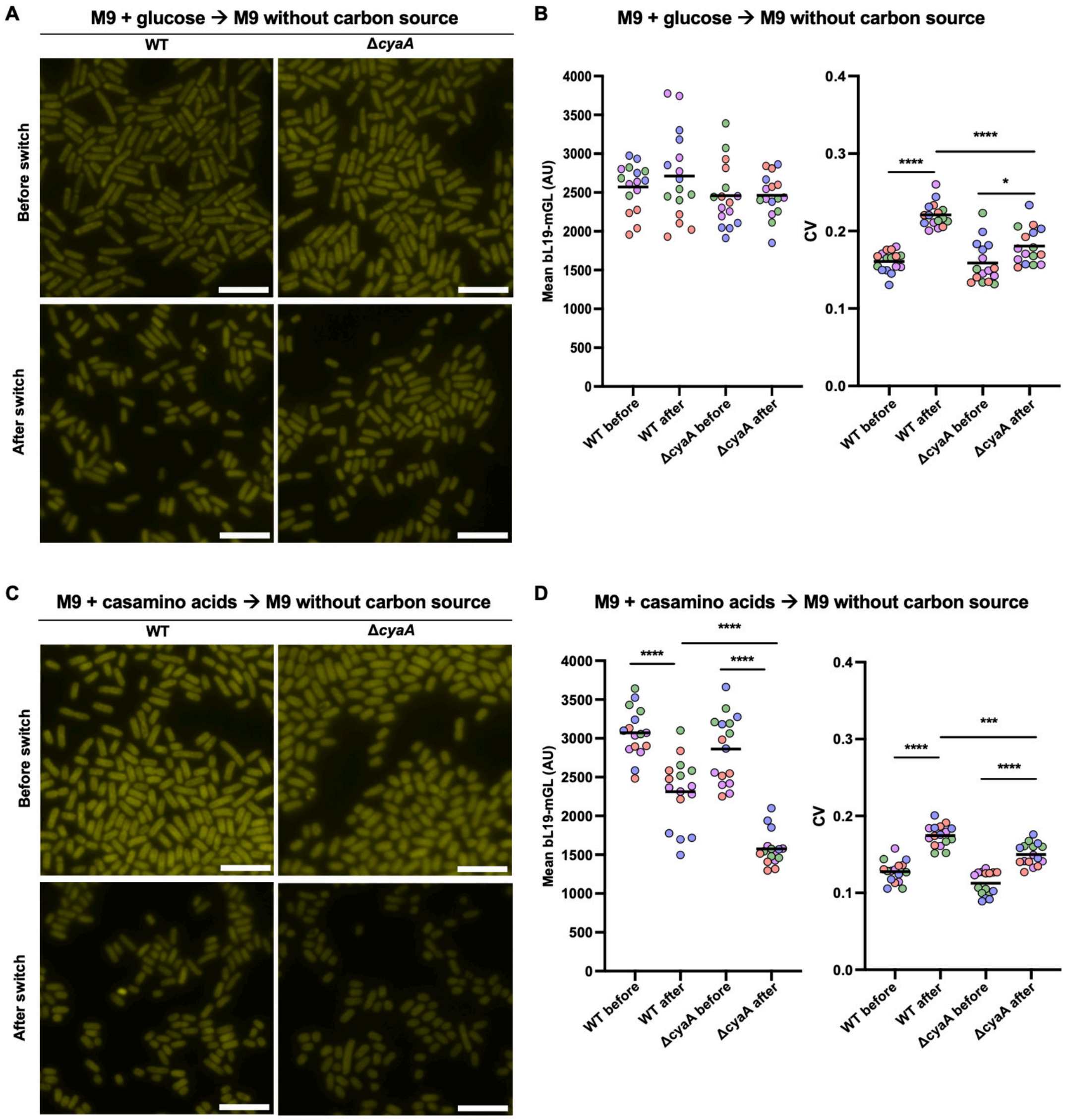

Fig. S28. Heterogeneity of RP levels upon carbon starvation, related to Fig. 9. WT and Δ*cyaA* bL19-mGL cells were grown in M9 with 0.4% glucose or casamino acids to mid log phase at 37°C, switched to M9 salts without any carbon source, and further incubated for 17 h prior to imaging. (A, C) Representative fluorescence images. (B, D) Means and heterogeneity of bL19-mGL fluorescence. Biological replicates are indicated in different colors, and technical replicates are shown in the same colors. The P values are calculated two-way ANOVA with Tukey's post-hoc test. * $P < 0.05$; *** $P < 0.001$; **** $P < 0.0001$. Scale bars: 5 μm.

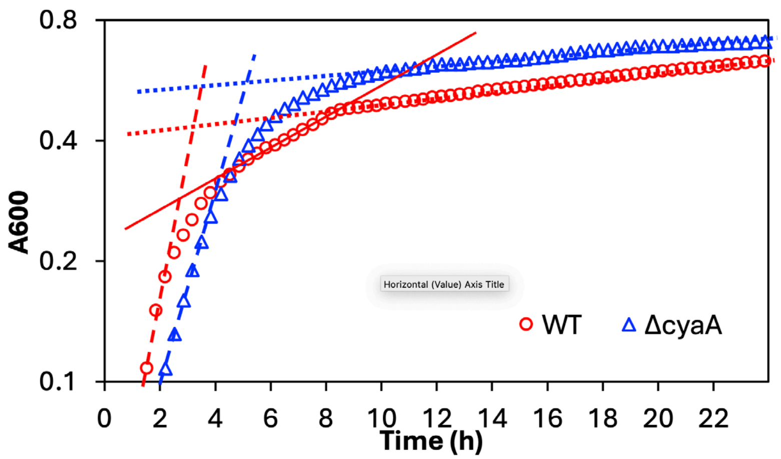

Fig. S29. Growth of *Salmonella* cells in LB at 37°C, related to Figs. 4 and 8. A600 over time for WT (circles) and Δ*cyaA* (triangle) cells inoculated from an overnight culture into fresh LB at time 0. Data are taken in the plate reader after subtracting the background. Each data point is an average of four biological replicates. The dashed lines indicate the initial exponential growth regime, which is faster for WT cells. The dotted lines indicate barely growing cultures 8~9 h after inoculation. While the Δ*cyaA* culture exhibits a broad transition from exponential growth from 4-8 h, the WT culture exhibited a distinct intermediate growth regime, indicated by the solid line. Linearity of A600 with cell density is shown in Fig. S20.

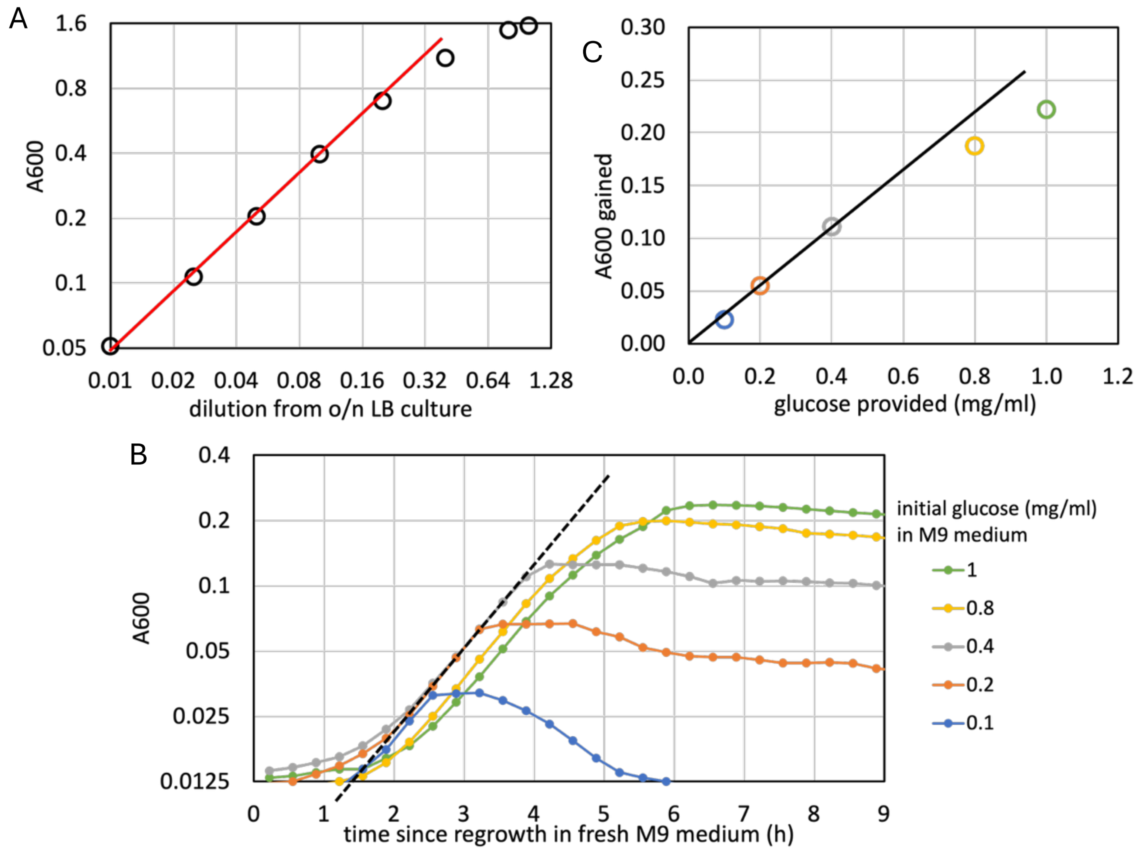

Fig. S30. Linearity and absolute calibration of optical density, related to Figs. 5 and 8. (A) Overnight LB culture of WT Salmonella cells was first concentrated and subsequently diluted up to 100x, and the optical density was taken for each dilution. Y-axis shows the A600 value after background subtraction for each dilution. The linearity of A600 reading with cell density (or biomass density) is seen up to A600 ≈ 0.8, which spans the entire range of A600 reading obtained for the growth curves in Fig. S19. (B) and (C): To provide an absolute calibration of the A600 reading, we grew WT Salmonella cells in M9 medium with glucose as the sole carbon source. Various amounts of glucose were used, from 0.1 mg/ml to 1 mg/ml. panel (B) shows the growth curve obtained in the plate reader for medium with each initial glucose concentration, after inoculation from an LB overnight culture. A600 reading is background-subtracted. After an initial lag of 1-2 h, each culture grew exponentially for a period, as indicated by the dashed black line (which indicates a doubling time of 45min). Cultures with different amounts of initial glucose plateaued at different A600 values. (C) The peak A600 value reached subtracted by the initial A600 at the time of inoculation, refers to as "A600 gained" (y-axis) and indicates the amount of biomass gained, is plotted against the initial glucose concentration in the medium. The relation is linear up to an initial glucose concentration of 0.5 mg/ml, with the black line indicating a slope corresponding to A600 ≈ 0.28 per mg/ml of glucose. (The tapering down of A600 at high glucose concentration likely reflects reduced yield as the culture becomes microaerobic.) We define the extrapolated A600 value reached by a culture with 1 mg/ml of glucose as 1 unit of common OD, since the biomass yield for growth on glucose is typically 0.5g CDW per g glucose, and 1 OD*ml of cells grown on glucose has 0.5 mg of cell dry weight (CDW) (*69*). Then, it follows that 1 unit of A600 on our plate reader corresponds to a common OD≈3.6. Using this absolute scale for A600 for the growth curves in Fig. S19, we see that the WT culture started to deviate from exponential growth at common OD≈0.7 (A600≈0.2), and experienced growth arrest at common OD≈1.7 (A600≈0.48).

Table S1. Key resources used in this study.

REAGENT or RESOURCE	SOURCE	IDENTIFIER
Antibodies		
Mouse anti-GFP	Roche	Cat#11814460001
Goat Anti-Mouse IgG-Peroxidase antibody	Invitrogen	Cat# 31430
Bacterial and virus strains		
Salmonella Typhimurium ATCC 14028s	ATCC	N/A
Salmonella bL19-mGL	This work	N/A
Salmonella bL19-mGL/P*tetA*-eCFP	This work	N/A
Salmonella bL19-mGL/uS2-eCFP	This work	N/A
Salmonella bL19-mGL/Cya-mCherry	This work	N/A
Salmonella uS2-mGL	This work	N/A
Salmonella Δ*cyaA*	This work	N/A
Salmonella Δ*dksA*	This work	N/A
Salmonella bL19-mGL Δ*cyaA*	This work	N/A
Salmonella bL19-mGL Δ*dksA*	This work	N/A
Salmonella bL19-mGL Δ*cyaA/dksA*	This work	N/A
Salmonella bL19-mGL Δ*crp*	This work	N/A
Salmonella bL19-mGL Δ*cpdA*	This work	N/A
Salmonella bL19-mGL Δ*relA/spoT*	This work	N/A
Salmonella bL19-mGL/P*tetA*-eCFP Δ*cyaA*	This work	N/A
Salmonella bL19-mGL/P*tetA*-eCFP Δ*dksA*	This work	N/A
Salmonella uS2-mGL Δ*cyaA*	This work	N/A
Salmonella uS2-mGL Δ*dksA*	This work	N/A
Salmonella uS2-mGL Δ*cyaA/dksA*	This work	N/A
Salmonella bL19-mGL Δ*rmf*	This work	N/A
Salmonella bL19-mGL Δ*raiA*	This work	N/A
Salmonella bL19-mGL Δ*rmf/raiA*	This work	N/A
E. coli K12 MG 1655	Lab collection	N/A
MG 1655 bL19-mGL	This work	N/A
Chemicals, peptides, and recombinant proteins		
Xpert protease inhibitor cocktail (100X)	GenDepot	Cat# P3100-010
Q5 High Fidelity 2X Master Mix	NEB	Cat# M0491L
Taq Polymerase 2X Master Mix	Apex Bioresearch Products	Cat# 42-133
Ampicillin sodium sulfate	Fisher Bioreagents	Cat# 69-52-3
Chloramphenicol	Sigma-Aldrich	Cat# 56-75-7
Spectinomycin	Sigma-Aldrich	Cat# 22189-32-8
Rifampicin	Chem-Impex	Cat# 00260
Adenosine-3',5'-cyclic monophosphate(cAMP)	Thermo Fisher Scientific	Cat# 60936.06
Spectinomycin dihydrochloride pentahydrate	Sigma-Aldrich	Cat# S9007
Critical commercial assays		
Plasmid Miniprep Kit	Zymo Research	Cat# D4019
PCR & DNA Cleanup Kit	Zymo Research	Cat# D40005
Click-iT™ Plus OPP Alexa Fluor 594 Protein Synthesis Assay Kit	Thermo Fisher Scientific	Cat# C10457
iScript cDNA Synthesis Kit	Bio-Rad	Cat# 170-8891
Low-ROX qPCR Mix	BioLink Laboratories	Cat# 16-2000-L
Clarity Western ECL Blotting Substrate	Bio-Rad	Cat# 1705060
Pierce BCA Protein Assay Kit	Thermo Fisher Scientific	Cat# 23225
Deposited data	ProteomeXchange Consortium	PXD064563

Continued

REAGENT or RESOURCE	SOURCE	IDENTIFIER
Oligonucleotides		
Primers for RT-qPCR	IDT	See Table S4
Primers for knockouts	IDT	See Table S4
Primers for recombinant DNA	IDT	See Table S4
Recombinant DNA		
pZS-P*tetA*-eCFP	This work	N/A
pZS-P*tetA*-eCFP-Chl-FRT	This work	N/A
REAGENT or RESOURCE	SOURCE	IDENTIFIER
Recombinant DNA		
pZS-P*tetA*-eCFP	This work	N/A
pZS-P*tetA*-eCFP-Chl-FRT	This work	N/A
pZS-P*araBAD*-mCherry	This work	N/A
pZS-P*tetA*-eCFP-P*araBAD*-mCherry	This work	N/A
pZS-tet-m-P*rpsB*-eCFP	This work	N/A
p*ZS*-P*tet*-mGL	This work	N/A
p*ZS*-P*tet*-eCFP	Lab collection	N/A
p*ZS*-P*tet*-mCherry	Lab collection	N/A
p*ZS*-P*cyaA*-CyaA	This work	N/A
p*ZS*-P*dksA*-DksA	This work	N/A
pKD46	(*76*)	N/A
pKD3	(*76*)	N/A
pCP20	(*76*)	N/A
Software and algorithms		
Fiji	(*79*)	https://imagej.net
GraphPad Prism	GraphPad	https://www.graphpad.com
Cellpose	(*80, 81*)	https://github.com/mouseland/cellpose
Jupyter-lab	Project Jupyter	https://jupyterlab.readthedocs.io/

Table S2. Oligonucleotides used in this study.

Table S3. Proteomics analysis with tandem mass tags isobaric labeling (Excel file).

Table S4. Total abundance of protein groups (Excel file).
